# Best Practices for Building and Supporting Effective ACGME-Mandated Program Evaluation Committees

**DOI:** 10.15766/mep_2374-8265.11039

**Published:** 2020-12-10

**Authors:** Jessica Greenfield, Elias I. Traboulsi, Krista Lombardo-Klefos, S. Beth Bierer

**Affiliations:** 1 Postdoctoral Fellow in Medical Education, Center for Educational Resources, Cleveland Clinic; Clinical Instructor, Cleveland Clinic Lerner College of Medicine of Case Western Reserve University; 2 Graduate Medical Education Director and Professor, Department of Ophthalmology, Cole Eye Institute, Cleveland Clinic; 3 Graduate Medical Education Accreditation Administrator, Cleveland Clinic; 4 Director of Assessment and Evaluation and Associate Professor, Cleveland Clinic Lerner College of Medicine of Case Western Reserve University

**Keywords:** Evaluation, Accreditation, Program Evaluation, Faculty Development, ACGME, Residency, Program Evaluation Committee, Annual Program Evaluation, Flipped Classroom

## Abstract

**Introduction:**

The Accreditation Council for Graduate Medical Education (ACGME) mandates that residency training programs form program evaluation committees (PECs) to monitor program delivery and outcomes, generate annual program evaluations (APEs), facilitate strategic planning, and implement continuous quality improvement projects. Though PECs provide essential documentation to position programs for successful accreditation decisions, few resources exist in the literature for PEC members.

**Methods:**

Employing Kern's model for curriculum development, we conducted a needs assessment in 2016 that resulted in adding a 2-hour workshop on building and supporting effective PECs to a certificate program for residency program directors. The workshop used a flipped classroom model with prework readings and guiding questions to familiarize participants with ACGME requirements for PECs and APEs. Several activities helped participants identify best practices for PECs and discuss authentic examples of mission statements, APEs, and action plans.

**Results:**

From 2017 to 2019, we offered this workshop on three different occasions to a total of 42 participants (34 residency program directors/associate program directors and eight program coordinators). In 2019, 14 participants completed a web-based evaluation after the session. All agreed or strongly agreed that the workshop met the learning objectives, utilized interactive teaching methods, included useful APE examples, and provided valuable resources.

**Discussion:**

This workshop addresses a gap in the literature by helping program directors identify best practices for PECs. The APE template and workshop examples can be adjusted to fit the needs of individual institutions.

## Educational Objectives

By the end of this activity, learners will be able to:

1. Describe the role of the program evaluation committee (PEC) in continuous quality improvement, strategic planning, and ACGME accreditation.
2. Discuss roles of PEC members and others in monitoring and documenting graduate medical education program activities and outcomes.
3. Identify best practices of highly functioning PECs.
4. Discuss how PECs can use the annual program evaluation process and strategic planning to initiate and sustain continuous quality improvement.

## Introduction

The Accreditation Council for Graduate Medical Education (ACGME) requires residency program directors (PDs) to appoint program evaluation committees (PECs) with the charge to conduct and document annual program evaluations (APEs) for continuous quality improvement.^1^ PECs play a critical role by monitoring training programs' processes and outcomes and recommending action plans for faculty consideration. Additionally, PECs document program improvements, review program mission statements, and engage in strategic planning. All of these activities, when done well, foster program improvement and curricular innovation and provide essential evidence to prepare graduate medical education (GME) training programs for 10-year accreditation site visits.^2,3^

Given the importance of PECs, it is surprising that few resources exist to assist PEC members. For instance, the ACGME's Common Program Requirements offer limited guidance on how PECs should function beyond listing PEC composition and residency program elements to review.^1^ While the medical education literature provides guidance on how to conduct program evaluations,^4–8^ limited resources offer best practices for PECs in GME.^9^ The resources that do exist may not reflect current accreditation requirements or are difficult to access.^10^ We could locate no PEC materials in *MedEdPORTAL* and doubt whether busy clinicians would have time to review excellent textbook-length resources on program evaluation used in higher education.^11–13^ The dearth of existing PEC resources underscores the need to develop targeted resources and training, especially as PEC members typically consist of clinical faculty and residents who may not have formal experience with program evaluation methods or who may not appreciate the importance of APEs for program improvement and accreditation purposes. To address this gap, we developed a 2-hour workshop to orient PDs to PECs and discuss best practices for APEs.

Using Kern's model for curriculum development,^14^ we conducted a needs assessment in 2016 with residency PDs at our institution in order to identify gaps and strengths within the existing Program Directors' Certificate Program (PDCP). This yearlong program consists of 15 workshops that occur monthly on our main campus. The needs assessment revealed that PDs desired guidance with program evaluation as well as more interactive, skills-based sessions in the overall PDCP curriculum. In response, we added a new session titled Best Practices for Building and Supporting Effective ACGME-Mandated Program Evaluation Committees to this workshop series. During workshop planning, we met with personnel in the Office of Graduate Medical Education to identify institutional policies about PECs and APEs, and we reviewed the ACGME's Core Program Requirements.^1^ These efforts helped us identify some of the challenges GME personnel encountered when helping residency PDs appoint PECs and develop APEs. For instance, we learned that confusion existed about who should serve on PECs. In relation to APEs, several programs submitted written reports to our GME office that lacked clear mission statements, strategic plans, or detailed action plans even though these elements existed on the institution's structured APE template. Finally, we discovered a paucity of resources on PECs in the literature, making it important for us to adopt an interactive format to solicit participants' experience with PECs and provide skill-building activities.

## Methods

### Target Audience/Setting

We implemented this flipped classroom workshop on three different occasions (2017–2019) within the PDCP and used participants' questionnaire feedback to make improvements. Our target audience included PDs, associate PDs (APDs), and GME personnel at our institution. In 2019, we invited program coordinators (PCs) to participate in the workshop as well. We chose a flipped classroom model to engage participants and provide opportunities for large- and small-group discussion. The workshop took place in a classroom setting with rectangular tables and movable chairs.

### Workshop Delivery

Participants received preworkshop required readings (see the recommended reading in Appendix A) a week before the session. These readings provided ACGME requirements for PECs and APEs. The facilitator utilized the workshop guide (Appendix A) to deliver the workshop. We recommend using a conference room with a projector to display the PowerPoint presentation (Appendix B). If conducting the workshop face-to-face, round tables are desirable to encourage small-group discussion. We also suggest having a flip chart, whiteboard, or chalkboard to note key ideas for group viewing. For a time line of the workshop, please refer to the facilitator guide.

The workshop began with introductions (10 minutes). We solicited participants' experience with PECs during this time and distributed instructional materials (Appendices B–H). The next 5 minutes provided foundational material about workshop objectives, PECs, and the accreditation cycle.

We devoted 15 minutes to Activity 1 (Appendix C) in which the facilitator asked participants to pair up to discuss the questions in the activity and then report to the larger group. We set aside 5–7 minutes to debrief after this pair-and-share activity.

After briefly reviewing the features of program aims, presented in the PEC Best Practices Presentation (Appendix B), the facilitator directed the group to begin Activity 2 (Appendix D) where participants reviewed and discussed program aims in two APE examples (Appendices F and G). The small groups discussed aim examples for 5 minutes before convening for large-group discussion.

Activity 3 (Appendix E) continued to utilize the two APE examples (Appendices F and G). We allocated 20–25 minutes for this activity, which was completed in groups of three or four participants. Each small group, after reviewing the examples and completing the checklist, reported key points to the large group.

The final portion of the workshop allotted 10 minutes for participants to discuss tools used to generate an APE and review the standard APE template (Appendix H). The facilitator used the remaining time to solicit participants' questions and take-home messages. We then provided 5 minutes for participants to complete the feedback questionnaire (Appendix I).

### Data Collection

In 2019, we asked participants to complete an electronic feedback questionnaire at the close of the session via the learning management system used for this longitudinal series. The questionnaire contained Likert-scale as well as open-ended questions (Appendix I). In prior years, participants evaluated the PDCP as a whole through focus groups, rather than completing individual workshop evaluations, thereby explaining the limited, specific feedback for this workshop. Our institutional IRB categorized the workshop evaluation form as a continuous quality improvement project.

### Data Analysis

We aggregated participants' ratings for Likert-scale questions and computed descriptive statistics. Content analysis of responses to open-ended questions helped us identify major themes.

## Results

To date, more than 170 PDs, APDs, and PCs have participated in the PDCP. Of these participants, 42 (34 PDs and eight PCs) have attended the workshop's three offerings.

In the 2019 iteration, 14 participants completed a workshop questionnaire and provided positive ratings about the workshop. For instance, all participants agreed the workshop met the learning objectives, utilized interactive teaching methods, and provided valuable resources. Additionally, all participants agreed the small-group discussions and APE examples were useful components of the workshop.

Participants' responses to open-ended questions included comments such as “I better comprehend the role and issues regarding the program evaluation committee” and “The example of an excellent program evaluation form was very helpful to use as a guide.” Others noted, “A structured committee with standing members and rotating members was a good idea I will try to implement” and “Having model documents to model our own behavior is of great utility.” A compilation of questionnaire feedback can be found in the Table.

**Table.**
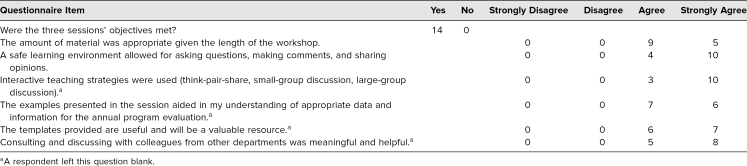
Participants' Feedback About the 2019 Workshop (*n* = 14, 100% Response Rate)

## Discussion

Tasked with reviewing and evaluating residency training programs, PECs play a critical role in GME. PECs monitor training program delivery, effectiveness, and outcomes. Additionally, PECs generate APEs that include recommendations to improve residency training program processes and outcomes. Highly functioning PECs contribute to continuous quality improvement activities and provide essential documentation for ACGME accreditation. We developed this workshop in response to our residency PDs' desire for guidance about PECs and program evaluation.

We offered this workshop on three different occasions and received positive feedback from participants. The workshop appeared to achieve the desired learning objectives, based on participants' ratings, and addressed a gap identified in the certificate program's needs assessment. We were successful with facilitating an interactive session, and workshop attendees actively participated in both large- and small-group discussions. Qualitative comments revealed that participants appreciated discussing best practices for PECs, such as having standing members serve on the committee, and reviewing authentic examples of APEs. We added SWOT (strengths, weaknesses, opportunities, threats) analysis to the workshop presentation and updated APE examples to illustrate superficial and detailed approaches to program evaluation. We also invited PCs to participate in the workshop as these individuals often serve on PECs or assist with APE completion. Locating up-to-date prereadings remains a challenge. To help address this gap, we plan to write a manuscript on PECs and APEs, using a 12-tip format, to add to the workshop materials. We have committed to offering the workshop virtually this coming year due to social distancing requirements. Zoom may serve as the best platform to support small-group discussions through the use of breakout rooms.

Our workshop has a few limitations. We delivered this session on the main campus of a large institution hosting a yearlong certificate program for PDs and APDs. Thus, participants may reflect a self-selected group interested in career development who could negotiate for release time from clinical responsibilities to attend. Our workshop evaluation collected only participant satisfaction data and did not include intermediate or long-term outcomes. Additionally, we did not have a session-specific evaluation for the first two offerings of the workshop as the leadership team for the certificate program obtained focus group feedback from certificate recipients at the end of the program. We received IRB approval to conduct two focus groups with program participants at different stages in the accreditation process prior to the COVID pandemic. Our aim was to explore the extent to which the workshop and structured APE template helped PDs appoint PECs, generate written APEs, and prepare accreditation self-studies. We have delayed these focus groups until the COVID crisis subsides.

We believe this workshop addresses a gap in the literature in that it helps PDs identify best practices for PECs and discuss authentic examples of APEs. We received positive feedback about workshop activities and believe this workshop is appropriate for any institution with ACGME training programs. Furthermore, the APE template used in workshop examples can be adjusted to fit the needs of individual institutions, which GME offices can use to help PDs generate APEs and standardize APE documentation across departments.

## Appendices

Facilitator Guide for PEC Workshop.docxPEC Best Practices Presentation.pptActivity 1 Pair-and-Share.docxActivity 2 Small-Group Discussion of Aims.docxActivity 3 Small-Group Discussion of Data Sources.docxAPE Weak Example.pdfAPE Strong Example.pdfAPE Template With Notes.docSession Evaluation Form.docx
All appendices are peer reviewed as integral parts of the Original Publication.
