# Supplementary material for: Best Practices for Building and Supporting Effective ACGME-Mandated Program Evaluation Committees
Source: MedEdPORTAL. 2020 Dec 10;16:11039. doi: 10.15766/mep_2374-8265.11039 (PMC7732133; doi:10.15766/mep_2374-8265.11039)
Supplement: Supplementary file 1 — Facilitator Guide for PEC Workshop.docxPEC Best Practices Presentation.pptActivity 1 Pair-and-Share.docxActivity 2 Small-Group Discussion of Aims.docxActivity 3 Small-Group Discussion of Data Sources.docxAPE Weak Example.pdfAPE Strong Example.pdfAPE Template With Notes.docSession Evaluation Form.docx [file mep_2374-8265.11039-s001.zip › A. Facilitator Guide for PEC Workshop.docx]

Best Practices for ACGME Mandated Program Evaluation Committees

Jessica Greenfield, PhD

Clinical Instructor, Internal Medicine

Postdoctoral Fellow in Medical Education

Education Institute, Cleveland Clinic

Cleveland, Ohio

Elias I. Traboulsi, MD, MEd

Professor, Ophthalmology

Chair, Department of Graduate Medical Education

Education Institute, Cleveland Clinic

Cleveland, Ohio

Krista Lombardo-Klefos, MBA

Accreditation Administrator

Department of Graduate Medical Education

Education Institute, Cleveland Clinic

Cleveland, Ohio

S. Beth Bierer, PhD, MEd

Associate Professor, Internal Medicine

Director of Assessment and Evaluation

Cleveland Clinic Lerner College of Medicine of Case Western Reserve University

Education Institute, Cleveland Clinic

Cleveland, Ohio

**Background and Description**

In 2012, the Accreditation Council for Graduate Medical Education (ACGME) approved the Next Accreditation System (NAS), whereby a 10-year review cycle was implemented to inform formal accreditation decisions involving ACGME residency and fellowship training programs. The NAS requires all ACGME programs to form Program Evaluation Committees (PECs), with specific membership requirements, in order to engage in and oversee ongoing quality improvement initiatives, as documented in ACGME-mandated annual program evaluation reports.

We observed that residency and fellowship program directors often lack formal training in program evaluation. Consequently, they may not appreciate how to recruit and prepare faculty, learners, and administrators to serve effectively on Program Evaluation Committees. This 2-hour workshop was designed to prepare key stakeholders in graduate medical education for their roles and responsibilities on PECs. Additionally, this workshop provides tools and examples to help PEC members generate actionable annual program evaluation reports and engage in strategic planning.

We implemented this 2-hour workshop on three separate occasions at the Cleveland Clinic and received positive feedback from program directors and graduate medical education administrators. Workshop participants especially appreciated “hands on activities”, authentic examples of program evaluation reports, and group discussion of best practices. This facilitator’s guide provides guidance on how to utilize workshop materials.

**Intended Audience**

This workshop is designed for residency program directors, faculty and residents serving on Program Evaluation Committees, and educational coordinators or administrators in graduate medical education.

**Workshop Objectives**

By the end of this workshop, participants will be able to:

- Identify requirements for Program Evaluation Committees.
- Discuss processes Program Evaluation Committee members can use in order to monitor, document, and improve training program activities and outcomes.
- Compare and contrast annual program evaluation reports using a template.
- Discuss best practices of highly functioning Program Evaluation Committees.

**Instructional Materials and Resources**

1. Access to conference room with computer in order to project workshop presentation, if session is delivered as a face-to-face workshop. Preferably the room will have round tables rather than rectangular tables and lecture-hall set up.
2. Participant access to the below materials, either as hard copies and posted to an electronic platform.
   1. PowerPoint presentation (Appendix B)
   2. Activity #1 handout (Appendix C)
   3. Activity #2 handout (Appendix D)
   4. Activity #3 handout (Appendix E)
   5. Example #1 of annual program evaluation report (weak) (Appendix F)
   6. Example #2 of annual program evaluation report (strong) (Appendix G)
   7. Annual Program Evaluation template with notes (Appendix H)

1. One flipchart and markers / whiteboard and markers / chalkboard and chalk

**Detailed Lesson Plan**

This workshop is designed as a face-to-face session that can be delivered in 1.5 or 2 hours, depending upon the number of participants. Some of these materials may be adapted as an online learning module, assuming participants could access a classroom management system and work in small groups synchronously with appropriate facilitation to guide group work and discussion.

| **Time** | **Activity** | **Appendix** | **Facilitator Role** |
| --- | --- | --- | --- |
| 10 minutes | **Introductions**   - Introduce self and orient participants to purpose of workshop. - Assess participants’ experience with program evaluation committees. | Packet of information:   - PEC Best Practices Presentation (Appendix B) – recommended as 3/page to allow for notetaking - Activity 1 Pair-and-Share (Appendix C) - Activity 2 Small Group Discussion of Aims (Appendix D) - Activity 3 Small Group Discussion of Data Sources (Appendix E) - APE Weak Example (Appendix F) - APE Strong Example (Appendix G) - APE Templates with Notes (Appendix H) | - Distribute packet of information. - Ask participants to use name cards. |
| 5 minutes | **Overview**   - Briefly review accreditation cycle - Present learning objectives - Define program evaluation committee | - PEC Best Practices Presentation (Appendix B) | - Display slides 1-7, using talking points and notes in slideshow to set stage |
| 15 minutes | **Activity 1 – Pair-and-Share**   - Ask participants to pair with another person (or 2 people if group has odd number of participants). - Have participants discuss questions on Activity #1 with colleague and be prepared to report to larger group. - Debrief in large group after 5-7 minutes for pair-share activity | - PEC Best Practices Presentation (Appendix B) - Activity 1 Pair-and-Share (Appendix C) | - Advance to slide 8. - Direct participants to handout for Activity #1. - Debrief afterward, noting key points on flipchart if available. |
| 5-10 minutes | **Review Aims**   - Review features of aims and rationale for generating aims. - Ask large group about their perceptions of aims they reviewed in two examples. | - PEC Best Practices Presentation (Appendix B) - Activity 2 Small Group Discussion of Aims (Appendix D) - APE Weak Example (Appendix F) - APE Strong Example (Appendix G) | - Direct participants to handout for Activity #2. - Advance to slide 13. - Move around room to monitor conversation. - Advance to slide 14. - Debrief. |
| 20-25 minutes | **Activity #3 – Small group Discussion**   - Ask participants to form small groups of 3-4 members (probably same groups for previous work). - Have participants examine both examples of program evaluation reports and complete checklist and guiding questions on handout for Activity #3 - Ask each group to report on key points for large group after they have spent about 15-20 minutes reviewing examples. | - PEC Best Practices Presentation (Appendix B) - Activity 3 Small Group Discussion of Data Sources (Appendix E) - APE Weak Example (Appendix F) - APE Strong Example (Appendix G) | - Advance to slide 17 and explain importance of data to inform decisions. - Direct participants to handout for Activity #3 and orient them to two examples of annual program evaluation reports. - Advance to slide 18. - Move around room to monitor conversation. - Advance to slide 19. - Debrief. |
| 7-10 minutes | **Review Tools**   - Review PDSA cycle and SWOT. - Solicit participants’ experience with PDSA or connect to one of examples. | - PEC Best Practices Presentation (Appendix B) | - Present key points outlined on slides 20-22. - Debrief examples. |
| 10 minutes | **Wrap Up and Evaluation**   - Solicit participants’ take home messages, best practices, and/or pearls. - Workshop evaluation | - PEC Best Practices Presentation (Appendix B) - Session Evaluation Form (Appendix I) | - Ask about take home messages/pearls. Post on flip chart. - Ask participants to complete workshop evaluation. Collect at end of session. |

**Evaluation**

We include an evaluation form in workshop materials that participants can complete at the end of the session.

**Facilitator Qualifications**

One facilitator can conduct this workshop. This person should possess the following qualifications:

- Knowledge of ACGME requirements for program evaluation committees, annual program evaluations, and accreditation site visits.
- Experience with graduate medical education.
- Ability to facilitate large and small group discussions effectively.
- Familiarity with all workshop materials as well as recommended resources listed in this guide.

We also recommend inviting one or more representatives from the Department of Graduate Medical Education if this department provides institutional oversight of accredited graduate medical education training programs.

**Recommended Resources**

Recommended reading:

- David A. Cook (2010) Twelve tips for evaluating educational programs, Medical Teacher, 32:4, 296-301, DOI: 10.3109/01421590903480121
- David W. Musick (2006) A Conceptual Model for Program Evaluation in Graduate Medical Education, Academic Medicine, 81:8, 759-765
- Simpson, D., & Lypson, M. (2011). The Year is Over, Now What? The Annual Program Evaluation. *Journal of graduate medical education*, *3*(3), 435–437. doi:10.4300/JGME-D-11-00150.1
- Guralnick, S., Hernandez, T., Corapi, M., Yedowitz-Freeman, J., Klek, S., Rodriguez, J., … Wade, L. (2015). The ACGME Self-Study-An Opportunity, Not a Burden. *Journal of graduate medical education*, *7*(3), 502–505. doi:10.4300/JGME-D-15-00241.1
- The Annual Program Evaluation, Self-Study, and 10-Year Accreditation Site Visit: Connected Steps in Facilitating Program Improvement. (2017). *Journal of graduate medical education*, *9*(1), 147–149. doi:10.4300/JGME-D-17-00047.1
- Amedee RG, Piazza JC. Institutional Oversight of the Graduate Medical Education Enterprise: Development of an Annual Institutional Review. *Ochsner Journal*. 2016;16(1):85-89.

Recommended Links:

Link describing NAS: <https://jcesom.marshall.edu/media/19073/NAS_FAQ-.pdf>

Link to ACGME Common Program Requirements: <https://www.acgme.org/Portals/0/PFAssets/ProgramRequirements/CPRResidency2019.pdf>

SWOT Analysis and Program aims resources:

- <https://www.mindtools.com/pages/article/newTMC_05.htm>
- Teoli D, An J. SWOT Analysis. [Updated 2019 Jan 4]. In: StatPearls [Internet]. Treasure Island (FL): StatPearls Publishing; 2019 Jan-. Available from: <https://www.ncbi.nlm.nih.gov/books/NBK537302/>
- <https://www.acgme.org/Portals/0/PDFs/Webinars/Webinar%20Handout.docx?ver=2018-09-06-134832-867>
- <http://www.acgme.org/Portals/0/PDFs/Webinars/DIOWebinarSelfStudy03112015.pdf>
- <https://www.acgme.org/Portals/0/PDFs/SelfStudy/SSAimsIPLK.pdf>
