# Supplementary material for: Best Practices for Building and Supporting Effective ACGME-Mandated Program Evaluation Committees
Source: MedEdPORTAL. 2020 Dec 10;16:11039. doi: 10.15766/mep_2374-8265.11039 (PMC7732133; doi:10.15766/mep_2374-8265.11039)
Supplement: Supplementary file 1 — Facilitator Guide for PEC Workshop.docxPEC Best Practices Presentation.pptActivity 1 Pair-and-Share.docxActivity 2 Small-Group Discussion of Aims.docxActivity 3 Small-Group Discussion of Data Sources.docxAPE Weak Example.pdfAPE Strong Example.pdfAPE Template With Notes.docSession Evaluation Form.docx [file mep_2374-8265.11039-s001.zip › C. Activity 1 Pair-and-Share.docx]

**Activity 1: Pair-and-Share about Program Evaluation Committee Membership and Functioning**

**Pair with another person, and discuss the following questions:**

- Who should serve on the Program Evaluation Committee based on your experience? For how long?
- How do you recruit members? What are their roles? How can these different members contribute to the Program Evaluation Committee?
- How should the Program Evaluation Committee chair orient members to their roles?
- What best practices have you observed? What challenges have you encountered?
- How often and when should the Program Evaluation Committee meet?
