# Supplementary material for: Best Practices for Building and Supporting Effective ACGME-Mandated Program Evaluation Committees
Source: MedEdPORTAL. 2020 Dec 10;16:11039. doi: 10.15766/mep_2374-8265.11039 (PMC7732133; doi:10.15766/mep_2374-8265.11039)
Supplement: Supplementary file 1 — Facilitator Guide for PEC Workshop.docxPEC Best Practices Presentation.pptActivity 1 Pair-and-Share.docxActivity 2 Small-Group Discussion of Aims.docxActivity 3 Small-Group Discussion of Data Sources.docxAPE Weak Example.pdfAPE Strong Example.pdfAPE Template With Notes.docSession Evaluation Form.docx [file mep_2374-8265.11039-s001.zip › D. Activity 2 Small-Group Discussion of Aims.docx]

**Activity 2: Small Group Discussion of Program Aims**

**In small groups, review the two examples below and discuss:**

- **How does your group rate the examples? Which is superior? Why?**
- **What should be included in a program’s aims statement?**
- **How should a training program’s aims inform the APE process?**

**Program 1**

To train the finest -- in the world. We seek to send out of our program trainees who are:

1. Intellectually curious, who will continue to approach their craft with a desire to remain committed to life-long learning.
2. Technically superior, with a vast array of skills.
3. Dedicated to continuous improvement in quality, safety and knowledge base.
4. Professional in their approach to patient care, interactions with co-workers, appearance, and obligations of their practice environment.

**Program 2**

Our goal is to train a well-rounded pathologist who can: 1) act as an effective diagnostic and clinical consultant; 2) critically assess medical literature and research; and 3) function independently without direct supervision as a valued physician in any setting.

To accomplish this goal, our program offers a wealth of clinical material, an outstanding subspecialty faculty, and up-to-date facilities in a structured, but flexible, educational environment in order to prepare residents to obtain certification by the American Board of Pathology. Core rotations in Anatomic, Clinical and Molecular Pathology are augmented by elective opportunities that encourage development of subspecialty expertise. Research activity is encouraged, and many opportunities are available.

We strive to maintain an educational and working environment in which concerns of the residents are addressed in a confidential and protected manner. Appropriate educational resources are provided, including a wide variety of case material, staff supervision, and information technology and reference materials. Residents are encouraged to participate in research activities including presentations at regional and national meetings and publication of their findings in scholarly journals. Opportunities for professional development as a teacher are provided through educational seminars (REALL program) and practical experience by presenting at conferences and Grand Rounds. A culture of professionalism supports patient safety and personal integrity. The educational environment includes graded and progressive clinical responsibility that fosters the development of a level of competence sufficient to practice without direct supervision upon completion of the program.
