# Supplementary material for: Best Practices for Building and Supporting Effective ACGME-Mandated Program Evaluation Committees
Source: MedEdPORTAL. 2020 Dec 10;16:11039. doi: 10.15766/mep_2374-8265.11039 (PMC7732133; doi:10.15766/mep_2374-8265.11039)
Supplement: Supplementary file 1 — Facilitator Guide for PEC Workshop.docxPEC Best Practices Presentation.pptActivity 1 Pair-and-Share.docxActivity 2 Small-Group Discussion of Aims.docxActivity 3 Small-Group Discussion of Data Sources.docxAPE Weak Example.pdfAPE Strong Example.pdfAPE Template With Notes.docSession Evaluation Form.docx [file mep_2374-8265.11039-s001.zip › E. Activity 3 Small-Group Discussion of Data Sources.docx]

**Activity 3: Small Group Discussion of Data Sources**

**In small groups, review the two examples of Annual Program Evaluation reports and discuss:**

- **What data did you see cited in each Annual Program Evaluation report?**

| **Example 1** | **Example 2** |
| --- | --- |
| 🞎 Review and current status of Action Items identified in the last Annual Program Evaluation  🞎 Program Goals and Objectives  🞎 Assessment of previous curriculum changes  🞎 Assessment tools (evaluations)  🞎 Faculty development activities/needs  🞎 Summary of faculty evaluations–development needs, areas for improvement  🞎 Faculty scholarly activities  🞎 Trainee scholarly activities  🞎 Recruitment and retention of faculty and trainees  🞎 Trainee match results  🞎 Patient satisfaction surveys (if applicable)  🞎 Results of annual CC trainee program evaluation  🞎 Results of annual CC faculty program evaluation  🞎 Results of ACGME Resident Survey  🞎 Results of ACGME Faculty Survey  🞎 Results of previous Program Improvement Plan (PIP)  🞎 Previous RRC Notification Letters or Communications  🞎 Trainee performance  🞎 Certification exams  🞎 Aggregate data from formative assessments  🞎 In-service exam scores  🞎 Alumni surveys (*when available)*  🞎 Other:______________________ | 🞎 Review and current status of Action Items identified in the last Annual Program Evaluation  🞎 Program Goals and Objectives  🞎 Assessment of previous curriculum changes  🞎 Assessment tools (evaluations)  🞎 Faculty development activities/needs  🞎 Summary of faculty evaluations–development needs, areas for improvement  🞎 Faculty scholarly activities  🞎 Trainee scholarly activities  🞎 Recruitment and retention of faculty and trainees  🞎 Trainee match results  🞎 Patient satisfaction surveys (if applicable)  🞎 Results of annual CC trainee program evaluation  🞎 Results of annual CC faculty program evaluation  🞎 Results of ACGME Resident Survey  🞎 Results of ACGME Faculty Survey  🞎 Results of previous Program Improvement Plan (PIP)  🞎 Previous RRC Notification Letters or Communications  🞎 Trainee performance  🞎 Certification exams  🞎 Aggregate data from formative assessments  🞎 In-service exam scores  🞎 Alumni surveys (*when available)*  🞎 Other:______________________ |

- **What data inform decision-making about program quality? Anything missing?**
