# Supplementary material for: Best Practices for Building and Supporting Effective ACGME-Mandated Program Evaluation Committees
Source: MedEdPORTAL. 2020 Dec 10;16:11039. doi: 10.15766/mep_2374-8265.11039 (PMC7732133; doi:10.15766/mep_2374-8265.11039)
Supplement: Supplementary file 1 — Facilitator Guide for PEC Workshop.docxPEC Best Practices Presentation.pptActivity 1 Pair-and-Share.docxActivity 2 Small-Group Discussion of Aims.docxActivity 3 Small-Group Discussion of Data Sources.docxAPE Weak Example.pdfAPE Strong Example.pdfAPE Template With Notes.docSession Evaluation Form.docx [file mep_2374-8265.11039-s001.zip › F. APE Weak Example.pdf]

Program Information

Academic Year	2018-2019
Program Name	[redacted]
Program Director	[redacted]
Percentage of Time PD dedicates to this program	10%
Associate Program Director(s)	
Percentage of Time APD(s) dedicates to this program	
Program Coordinator	[redacted]
Percentage of Time PC dedicates to this program	20%
Date of Last RC Site Visit/Self-Study Visit	9/27/2011
Date of Upcoming Self-Study Visit	10/01/2021
Current Accreditation Status	Continued Accreditation
Current Number of Citations	0
Current Number of Area of Improvement/Concerning Trend	0
Length of Program	2 years
# of Trainees per Year	4
Total # of Approved RC Positions	8
Total # of Approved CCF Positions	8
Program Evaluation Committee (PEC) Members	[redacted]
Date(s) of PEC Meeting(s) held this academic year	06/03/2019
Date of Annual Program Evaluation (APE)	06/03/2019
Date APE and Action Plan was reviewed and approved by Faculty	06/03/2019

Attendees at the Annual Program Evaluation Meeting (add/remove lines as necessary)

Name	***Title***
[redacted]	Program Director
[redacted]	Staff
[redacted]	Staff
[redacted]	Staff
[redacted]	Staff
[redacted]	Staff
[redacted]	Staff
[redacted]	Staff
[redacted]	Staff

What are the aims of this training program - what differentiates this program from others?

The [redacted] Section at Cleveland Clinic is a world leader in the field. We recruit from the best

general [redacted] fellows both within the general fellowship at CCF and from outside if appropriate. We aim to provide fellows an unparalleled education, both hands-on and academically. Our fellows are integrally involved in all [redacted] and we make sure that they are not only well trained to perform these procedures but also excel upon graduation. Whether they pursue community or academic based careers, they are usually seen as the leaders in their area and we strive to maintain this reputation.

Describe activities taken this academic year to further the aim

We are continuing the high level of procedural volume (up more than 10% this year), involvement in clinical trials of cutting-edge procedure and devices, and remain dedicated to hands-on training. We have also revamped our didactic curriculum in order to make sure that our fellows remain apprised of the historic and current data regarding the work that we do. Academic productivity remains among the highest in the entire Enterprise and our fellows are involved in the same. Overall, our faculty remain dedicated to training leaders in community and academic [redacted].

Annual Program Evaluation Discussion Items

Subject	***Parameter***	***Comments/Response/Plans for Improvement***
ACGME Annual Faculty Survey Results	100% Very Positive	
ACGME Annual Resident Survey Results	25% Positive<br>75% Very Positive	
ACGME Milestone Update		
ACGME RC Notifications & Reponses (if applicable)	Received continued accreditation on 2/6/2019.	N/A
Alumni Survey Results (if applicable)		N/A
Annual Faculty Evaluation of Program Results		Desire for more administration support
Annual Trainee Evaluation of Program Results		Desire for more/better didactics
Assessment Tools (Evaluations)		Feedback during and after procedures. Quarterly evaluations in MedHub.
Case Logs/Procedures		[redacted] database
Clinical Competency Committee		Meets twice a year. December and June<br>[redacted]
Faculty Development (list activities)		No specific development activity.
Faculty Scholarly Activity		List attached
Graduating Resident Information		[redacted]
Interprofessional Education		N/A

GMEC Approved 9/27/2013, 2/8/2019
Minor Edits: 4/2015, 3/2017, 1/2019

Subject	Parameter	Comments/Response/Plans for Improvement
Major Changes in the Program since last ADS update		N/A
Outside Rotators/Other Learners		N/A
Outstanding Program Accomplishments/Kudos		N/A
Program Goals & Objectives		Continue outstanding clinical training.<br>Continue research mentorship.
Program Match Results/Recruitment Efforts	N/A	N/A
Quality Improvement and Patient Safety Projects (include status)		Supervised machine learning to predict post PCI AKI / status abstract accepted / manuscript being written<br><br>Fast track STEMI / status just started
Research Curriculum		Fellow – ongoing
Results of last year's Program Improvement Plan (PIP)		Provided /Reviewed at meeting
Review of last year's Annual Program Evaluation (APE)		Provided / Reviewed at meeting
Trainee Performance on Board Exam (if applicable)		100% passed
Trainee Performance on In Service Exam (if applicable)	N/A	N/A
Trainee Scholarly Activity		Prolific
Well-Being		Excellent
Work Hours		Rare violations of 80hr/weeks; None when averaged over four weeks

Additional Discussion Items (add/remove lines as necessary)

Parameter	Comments/Response/Plans for Improvement
N/A	

Results of last 2 year's Annual Program Evaluation Action Plan(s)

	Areas for Improvement (AY 2016--17)	Intervention	Date instituted/ Individual responsible	Expected Resolution (outcome measures and date)	Status (resolved, partially resolved and detail, not resolved and date)
1	Re-iterate monthly feedback to the fellows as a requirement of the staff.	Program coordinator will compile MedHub evaluations. Fellows will follow up with staff monthly.	PD and Coordinator / July of 2017	December of 2017	Resolved Evaluations sent out quarterly.
2	Discuss directly with fellows if specific operators tend to be more troublesome to them with respect to procedural involvement and education more than others.	Survey monkey completed. One specific staff member identified who is no longer at CCF as of June 2017.	Resolved as the offending staff member left CCF; no further complaints on surveys re: respect of the fellows and procedural involvement.	Resolved as the offending staff member left CCF; no further complaints on surveys re: respect of the fellows and procedural involvement.	Resolved as the offending staff member left CCF; no further complaints on surveys re: respect of the fellows and procedural involvement.
3	Re-iterate to staff that their attendance at conferences is under their control.	Issue with survey where some staff thought the survey referred to general [redacted] conferences-staff is not expected to attend those.	Improvement in conference attendance as the staff now understand that the expected conference in the survey is only the [redacted] section one, not general fellowship. Also overall improved attending involvement in Friday AM conference	Improved attending involvement in Friday AM conference	Resolved
	**Areas for Improvement (AY 2017 - 18)**	**Intervention**	**Date instituted/ Individual responsible**	**Expected Resolution (outcome measures and date)**	**Status (resolved, partially resolved and detail, not resolved and date)**
1	Assess staff attendance at the fellows AM conferences	Will take weekly attendance	Attending involvement in the weekly conference (and in the didactic	Attending involvement in the weekly conference (and in the didactic curriculum) has not been an issue per the fellows and therefore	Resolved

GMEC Approved 9/27/2013, 2/8/2019
Minor Edits: 4/2015, 3/2017, 1/2019

			**curriculum) has not been an issue per the fellows and therefore formal attendance has not been taken.**	**formal attendance has not been taken.**	
2					

This Year's Annual Program Evaluation Action Plan(s)

Area for Improvement Identified	*Monitoring & Measuring Plan*
**Request for more/better didactics**	**Overhaul of didactic curriculum to begin July of 2019.**

Which aspects of Health Systems Science were incorporated into your curriculum during this academic year? *(Place an X in left column to denote; describe in right column)*

Subject Area		*Description*
☐	Clinical Informatics	
☒	Health Care Delivery System	[redacted] **models of care**
☐	Health Care Policy & Economics	
☐	Leadership in Healthcare	
☒	Patient Safety	**Reducing** [redacted]
☐	Population Health	
☒	Quality Improvement	**Improving** [redacted] **outcomes**
☐	Socio-ecologic Determinants	
☐	Teamwork & Team Science	
☐	Use of Assessments to Support Learning Improvement	
☐	Value in Health Care	

SWOT ANALYSIS - This will assist your program on preparing for the Self-Study, as you will need to use past APEs to develop a comprehensive summary of the program and its learning environment. A SWOT Guide and SWOT Analysis Template are located on GME|com.

Strengths	Weaknesses
Clinical volume is practically unparalleled.<br>Many/most faculty are world leaders in the field.<br>Academic productivity of the fellows and faculty is tremendous.<br>Dedication of the staff to the fellows as mentees and colleagues.	Criticism of Didactic curriculum instruction and attending involvement in the same; this has now been revised with very positive early feedback from the fellows.
**Opportunities**	**Threats**
Improvement of didactic curriculum as in the "Weaknesses" section.	None

GMEC Approved 9/27/2013, 2/8/2019
Minor Edits: 4/2015, 3/2017, 1/2019

Program Director Attestation – Typed signature below indicates that the APE content, or a synthesis of the APE, was reviewed and distributed to the program faculty:

[redacted] 06/05/2019

Please refer to the ACGME Common Program Requirement V.C. Program Evaluation and Improvement for additional information regarding the requirement.

Documentation to be used for the Annual Program Evaluation

- ☐ Review and current status of Action Items identified in the last Annual Program Evaluation
- ☐ Program Goals and Objectives
- ☐ Assessment of previous curriculum changes
- ☐ Assessment tools (evaluations)
- ☐ Faculty development activities/needs:
  - ☐ Summary of faculty evaluations–development needs, areas for improvement
  - ☐ Scholarly activities
- ☐ Recruitment and retention of faculty and trainees
- ☐ Trainee match results
- ☐ Patient satisfaction surveys (if applicable)
- ☐ Results of annual CC trainee program evaluation
- ☐ Results of annual CC faculty program evaluation
- ☐ Results of ACGME Resident Survey
- ☐ Results of ACGME Faculty Survey
- ☐ Results of previous Program Improvement Plan (PIP)
- ☐ Previous RRC Notification Letters or Communications
- ☐ Trainee performance:
  - ☐ Certification exams
  - ☐ Aggregate data from formative assessments
  - ☐ In-service exam scores
  - ☐ Scholarly activities
  - ☐ Alumni surveys (*when available)*
- ☐ Other:____

GMEC Approved 9/27/2013, 2/8/2019
Minor Edits: 4/2015, 3/2017, 1/2019
