# Supplementary material for: Best Practices for Building and Supporting Effective ACGME-Mandated Program Evaluation Committees
Source: MedEdPORTAL. 2020 Dec 10;16:11039. doi: 10.15766/mep_2374-8265.11039 (PMC7732133; doi:10.15766/mep_2374-8265.11039)
Supplement: Supplementary file 1 — Facilitator Guide for PEC Workshop.docxPEC Best Practices Presentation.pptActivity 1 Pair-and-Share.docxActivity 2 Small-Group Discussion of Aims.docxActivity 3 Small-Group Discussion of Data Sources.docxAPE Weak Example.pdfAPE Strong Example.pdfAPE Template With Notes.docSession Evaluation Form.docx [file mep_2374-8265.11039-s001.zip › G. APE Strong Example.pdf]

Annual Program Evaluation (APE)

Program Information

Academic Year	2018-2019
Program Name	[redacted]
Program Director	[redacted], MD
Percentage of Time PD dedicates to this program	50%
Associate Program Director(s)	[redacted], MD, [redacted], MD
Percentage of Time APD(s) dedicates to this program	40% [redacted], 30% [redacted]
Program Coordinator	[redacted], C-TAGME
Percentage of Time PC dedicates to this program	100%
Date of Last RC Site Visit/Self-Study Visit	12/13/2011
Date of Upcoming Self-Study Visit	10/1/2023
Current Accreditation Status	Continued Accreditation
Current Number of Citations	1
Current Number of Area of Improvement/Concerning Trend	0
Length of Program	4 years
# of Trainees per Year	Currently PGY1: 7; PGY2: 7; PGY3: 7; PGY4: 6
Total # of Approved RC Positions	36
Total # of Approved CCF Positions	28
Program Evaluation Committee (PEC) Members	See table below
Date(s) of PEC Meeting(s) held this academic year	4/3/2019, 4/17/2019, 4/24/2019, 5/1/2019, 5/8/2019
Date of Annual Program Evaluation (APE)	[redacted] 5/13/2019, [redacted] 5/20/2019
Date APE and Action Plan was reviewed and approved by Faculty	[redacted] 5/13/2019, [redacted] 5/20/2019

Members of the Annual Program Evaluation Committee

Name	***Title***
[redacted], MD	Residency Program Director, Committee Chair
[redacted], MD	Associate Program Director- [redacted]
[redacted], MD	Associate Program Director- [redacted]
[redacted], MD	Staff, [redacted]
[redacted], MD	Staff, [redacted]
[redacted], PhD	Staff, [redacted]
[redacted], MD	Staff, [redacted]
[redacted], MD	Resident Representative, PGY1 Class
[redacted], MD	Resident Representative, PGY1 Class
[redacted], MD	Resident Representative, PGY1 Class
[redacted], MD	Resident Representative, PGY2 Class
[redacted], MD	Resident Representative, PGY2 Class
[redacted], MD	Resident Representative, PGY3 Class

Annual Program Evaluation (APE)

[redacted], MD	Resident Representative, PGY4 Class

What are the aims of this training program - what differentiates this program from others?

Our goal is to train a well-rounded [redacted] who can
1. Act as an effective diagnostic and clinical consultant.
2. Critically assess medical literature and research.
3. Function independently without direct supervision as a valued physician in any setting.

To accomplish this goal, our program offers a wealth of clinical material, an outstanding subspecialty faculty, and up-to-date facilities in a structured, but flexible, educational environment in order to prepare residents to obtain certification by the American Board of [redacted]. Core rotations in [redacted], [redacted] and [redacted] are augmented by elective opportunities that encourage development of subspecialty expertise. Research activity is encouraged, and many opportunities are available.

We strive to maintain an educational and working environment in which concerns of the residents are addressed in a confidential and protected manner. Appropriate educational resources are provided, including a wide variety of case material, staff supervision, and information technology and reference materials. Residents are encouraged to participate in research activities including presentations at regional and national meetings and publication of their findings in scholarly journals. Opportunities for professional development as a teacher are provided through educational seminars (REALL program) and practical experience by presenting at conferences and Grand Rounds. A culture of professionalism supports patient safety and personal integrity.

The educational environment includes graded and progressive clinical responsibility that fosters the development of a level of competence sufficient to practice without direct supervision upon completion of the program.

Describe activities taken this academic year to further the aim

Please see annual program evaluation plan from 2017-2018 for last year's activities, and the plan from this year's APE for the planned interventions for the upcoming year.

Annual Program Evaluation Discussion Items

Subject/Data	*Comments/Response/Plans for Improvement*
**ACGME Annual Faculty Survey Results**	
The ACGME Faculty Survey was completed by 43/44 (98%) core faculty. Faculty's Overall Evaluation of the Program fell between "positive" (33%) and "very positive" (60%) and was ranked as slightly below the national average.<br><br>**Faculty Supervision and Teaching:**<br>Sufficient time to supervise residents/fellow: 84% (national average of 95%). Improvement from last year (74%).<br>Interest of faculty and Program Director in education: 88% (national average 97%). Improvement from last year (82%).<br><br>There was no significant difference in program and national compliance rates in "rotation and educational assignment evaluation", "residents/fellows seek	Overall, the program has seen improvements in many areas that were the focus of the program over the last 2 years (faculty engagement, QI/PS involvement, etc.). Although improved, faculty continue to feel that they have less time to supervise residents and fellows than the national average.<br><br>The faculty have a somewhat lower perception of the effectiveness of graduating residents/fellows compared to national data. It is unclear why this perception is lower. Possibilities include:<br>1. "Effectiveness" is somewhat unclear and may be in the eye of the beholder<br>2. AP faculty (majority of faculty) do not see residents in the PGY4 year, and thus are assessing on PGY3 performance<br>3. Many faculty (and residents) wish residents took more ownership of cases/responsibility, which may allow

Subject/Data	Comments/Response/Plans for Improvement
supervisory guidance", "faculty satisfied with personal performance feedback", and "faculty performance evaluated" between program and national average.<br><br>**Educational Content:**<br>Effectiveness of graduating residents/fellows: 81% compared to the national average of 98%. Improvement from last year (76%).<br><br>There were no significant differences in all the other sub-categories in terms of compliance rate or program mean score with national averages.<br><br>**Resources:**<br>There were no significant differences in all categories in terms of compliance rate or program mean score with national averages.<br><br>**Patient Safety:**<br>There were no significant differences in all categories in terms of compliance rate or program mean score with national averages.<br><br>**Teamwork:**<br>There were no significant differences in all categories in terms of compliance rate or program mean score with national averages.<br><br>**Important changes from the previous year include:**<br><br>There was improvement in "sufficient time to supervise residents/fellow" from 74% to 84%, although the figure is still significantly below the national average of 95%. "Faculty satisfied with personal performance feedback" also improved from 65% to 81%. The compliance rate for "residents/fellows participate in quality improvement or patient safety activities" also improved significantly from 80% to 90%. There were no significant decreases in compliance rates from the previous survey.	better assessment of their effectiveness. This issue is also important in the resident survey as well.<br><br>The program will consider creating a task force of faculty and residents to find ways to improve resident ability to take greater ownership of cases on [redacted]. There is good responsibility for cases in [redacted] already on lymphoma and hemepath.
**ACGME Annual Resident Survey Results**	
At the time of the Annual Program Review, the results of the 2018-2019 ACGME Resident Survey were not available, so the 2017-2018 Survey Results were reviewed. The survey was completed by 27/27 (100%) of residents. Highlights are summarized below: Overall Evaluation of the Program was between "positive (44%) and "very positive" (37%), with only 4% (1 individual) ranking the program as "negative". The program ranked slightly below the national average.<br><br>**Work Hours:**	The 2017-2018 survey showed continued stability or improvement in the areas of clinical experience and education, evaluation, resources, and patient safety/teamwork, which are areas of focus for the program for the last few years. In several areas the program is above the national mean:<br>1. "Satisfied that evaluations of the program are confidential"<br>2. "Satisfied that the program uses evaluations to improve"<br>3. "Residents can raise concerns without fear"<br>4. "Participated in quality improvement"<br><br>There continues to be issues with regards to service vs.

Subject/Data	Comments/Response/Plans for Improvement
There were no significant differences in all the sub-categories in terms of compliance rate or program mean score compared with national averages.<br><br>**Faculty:**<br><br>The program compliance rate for some categories were significantly below (>10%) the national mean, including “sufficient supervision” (81% versus national mean 92%), “sufficient instruction” (59% versus national mean 86%), “faculty and staff interested in residency education” (70% versus national mean 85%), and “faculty and staff create environment of inquiry” (56% versus national mean 79%). Additionally, one of these categories were significantly below the national mean the year before as well (“faculty and staff interested in residency education” - 74% one year ago). The category of “appropriate level of supervision” was in line with the national average.<br><br>**Evaluation:**<br><br>“Satisfied with feedback after assignments” was 56% (the same as the previous year), below the national average of 72%, whereas “satisfied that evaluations of program are confidential” and “satisfied that program uses evaluations to improve” were 96% and 85%, respectively, higher than the respective national averages of 87% and 75%. There was no significant difference in all the other subcategories between program and national averages.<br><br>**Educational Content:**<br><br>“Education (not) compromised by excessive reliance on non-physician obligations” was 41% (the same as the previous year), significantly below the national average of 75%. “Appropriate balance between education and other clinical demands” was 70% (this year as well as previous year) compared to the national average of 80%. There was no significant difference in all the other subcategories between program and national averages.<br><br>**Resources:**<br><br>“Residents can raise concerns without fear” (93% compliant) was significantly higher than the national average of 82%. There was no significant difference in all the other subcategories between program and national averages.<br><br>**Patient Safety/Teamwork:**<br><br>The program compliance rate for “tell patients of	education, in particular the ACGME metric of “Education (not) compromised by excessive reliance on non-physician obligations” (was 41% compliant responses). Discussion amongst the committee included evaluation of the following services:<br>1. [redacted] – Prior plan: [redacted] bench to allow greater review of [redacted] by residents. We continue to have issues with recruitment of [redacted] so with open positions this plan has not yet been implemented. Residents appreciate [redacted] covering lunch breaks this year. Will continue to advocate for this position once [redacted] positions are full. Additional measure are necessary to improve the experience until this happens.<br>2. [redacted] – this is a new concern, although it was brought up in the program-specific survey from June 2018 as well. Residents currently responsible for set up and cleaning of [redacted] (non-physician level work), but is this still appropriate? What is educational in July (review of every [redacted] in detail) is not necessarily educational in June. Could there be changes in which cases are presented at conference, or only show critical/educational [redacted]? There is a lot of repetition in preparation of [redacted] on multiple days. This will take careful discussion with [redacted] faculty.<br>3. [redacted] – Prior plan: Monitoring of on-call pages, with new protocols designed to reduce number of unnecessary pages. This has shown substantial improvement over time (PGY4s note significant changes). Continues to be issue of conference pages – 50% of time resident is paged out of conference when on TM. Previously discussed with [redacted] faculty; felt that additional handoff of pager too much risk for patient safety for 1 hr. Currently residents are excused from conference when on [redacted], and only accounts for 3 months of 4-year residency, with lecture series on 2-year repeat.<br>4. [redacted] – New concern in last year, but already handled with overflow service. Does not need intervention at this time.<br><br>There also continue to be issues with faculty (“sufficient instruction” and “faculty and staff create an environment of inquiry” and “satisfied with feedback after assignments”) although these have improved considerably over time. The program will continue the faculty development and engagement programs instituted over the last few years.

GMEC Approved 9/27/2013, 2/8/2019
Minor Edits: 4/2015, 3/2017, 1/2019

Subject/Data	Comments/Response/Plans for Improvement
respective roles of faculty and residents" was 89% compared to the national compliance rate of 98%. The compliance rate for "participated in quality improvement" was higher than the national average (96% vs 87%). There were no significant differences in all the other subcategories in terms of compliance rate or program mean score compared with national averages.<br><br>**Areas of Concern:**<br><br>PGY4 residents continue to rank the following areas below the national means: [redacted], [redacted], and [redacted].<br><br>**Summary:**<br><br>The survey was completed by 27/27 (100%) of residents. The overall evaluation of the program is slightly below the national mean, but with 81% rating as positive or very positive. Areas of concern remain for "faculty and staff create environment of inquiry" (56% currently vs 70% in 2016-2017) and "sufficient instruction" (59% currently vs 89% in 2016-2017). There was no change in compliant responses for "satisfied with feedback after assignments" and "education (not) compromised by excessive reliance on non-physician obligations" with 56% and 41% compliant responses, respectively, that are well below the national means. We remain 10% below national means for "sufficient supervision" "faculty and staff interested in residency education" and "appropriate balance between ed and other clinical demands".	
**ACGME Milestone Update**	
[redacted] Milestones 2.0 were released in February 2019, with an implementation date of July 1st, 2019. This is part of an overall update to unify Milestones of many different specialties by the ACGME. The new document incudes 21 Milestones (down from 27) and significantly simplified text. Now there are 19 [redacted] milestones and 3 [redacted]-only milestones. The target for graduation remains 4.<br><br>In the 2018-2019 annual faculty survey, 92% of faculty were familiar with the [redacted] Milestones.	The program is working to update all evaluation forms to reflect the Milestones 2.0 requirements, with an anticipated implementation date of July 1st, 2019. The new milestones have been distributed to all sub-specialty education coordinators to consider while updating evaluations. The new milestones have been distributed to all residents and discussed at our annual Resident Retreat on February 27th, 2019. We are awaiting update of MedHub with the new milestones to create the new evaluations.
**ACGME RC Notifications & Reponses (if applicable)**	
**EXTENDED CITATIONS**<br>**Service to Education Imbalance | Since: 02/04/2016 | Status: Extended**<br>Excessive Reliance on Residents to Fulfill Non-	The program is delighted to see the removal of 3 prior areas for improvement/concerning trends related to Educational Content, Evaluations, and Faculty Supervision and Teaching.

Subject/Data	Comments/Response/Plans for Improvement
Physician Service Obligations [CPR VI.A.4.b)]<br>The learning objectives of the program must not be compromised by excessive reliance on residents to fulfill non-physician service obligations. The information provided did not demonstrate compliance with the requirement. Resident survey responses over the past three years indicate resident perception that education is being compromised by service obligations. The Review Committee requests the program develop a plan to address any service issues that undermine residents' ability to participate fully in educational activities.<br><br>**Continued Non-Compliance: 01/21/2017**<br>The information provided did not demonstrate compliance with the requirement. Again, the Review Committee notes efforts made to resolve this citation; however, resident perception that service obligations do not compromise education remains well below the national mean for the category. The committee notes that the plan for improvement in the [redacted] areas was implemented; the plan for the [redacted] has not yet been implemented.<br><br>**Continued Non-Compliance: 01/29/2018**<br>The information provided to the Review Committee did not demonstrate substantial compliance with the requirement. The Review Committee notes the substantial efforts made by the program in working to improve this area, but the 2016-2017 Resident Survey still shows that resident perception remains that education may be being compromised by service obligations. The committee will continue to monitor this area in the next annual data review.<br><br>**Continued Non-Compliance: 01/14/2019**<br>The information provided to the Review Committee still did not demonstrate substantial compliance with the requirement, as the 2017-2018 ACGME Resident Survey indicated that 59 percent of residents felt their education was compromised by excessive reliance on non-physician obligations. The Committee noted the robust response to citations and efforts undertaken by the program to improve this issue; however, the Resident Survey has shown no improvement in this area for the past three years, and the Committee will continue to monitor until the survey shows demonstrated improvement.	The continued citation of Service vs. Education Imbalance remains a challenge for the program to improve. Substantial discussion regarding the issues around this perception occurred (see section on ACGME resident survey above).<br><br>Please see the action plan below for details on the plan for this year's work to improve in this area.
**Alumni Survey Results (if applicable)**	
The first-ever [redacted] alumni survey was sent to 80 alumni who completed training during 2009-2017 with a response rate of 49/80 (61.3%). The majority	The findings from the alumni survey validate the mission statement of the program, and it is heartening to see the strong alumni response rate and that all former residents have been

Subject/Data	***Comments/Response/Plans for Improvement***
completed training in the AP/CP track, and 47/49 (92%) completed one or more fellowships. Just over half of the fellowships were completed at Cleveland Clinic, with the three most popular areas including [redacted] (21), [redacted] (19) and [redacted] (8).<br><br>Our graduates are almost equally divided between academic (39%) and private practice (37%) settings, and the majority (69%) are still in their first "real" job. Most graduates have responsibilities in [redacted] (89%) and [redacted] (62.5%); more than 70% have administrative responsibilities in one or more areas. Most spend more of their time in [redacted] (86.5%) than in [redacted] (23.7%).<br><br>Based on their residency training at Cleveland Clinic, 43/45 (95.6%) of respondents felt they were adequately prepared for fellowship(s). Based on their experience in residency training, 35/45 (77.8%) and 27/45 (60.0%) felt they were adequately prepared for the "real world" practice of [redacted], respectively.<br><br>Three top areas in which graduates felt least prepared included [redacted] and billing, placental pathology, and chemistry. Although many varied responses were received, areas cited for better training included granting residents more responsibility for dictation, [redacted] and [redacted]; increasing time in [redacted], [redacted]; and more training in [redacted] and [redacted]. The majority of respondents would recommend (14/45; 31.1%) or strongly recommend (28/45; 62.2%) the Cleveland Clinic [redacted] Residency Program to others.	successful in acquiring jobs in the "real world". There is an equal division of alumni in private practice and academic practice. The alumni were generally happy with their training and the majority would recommend or strongly recommend the program to others.<br><br>The survey identified several areas for improvement, of which the program is most aware and actively working to change or was changed in the recent past. Opportunities for improvement not yet changed include:<br>1. [redacted] experiences<br>2. Increased responsibility for cases by residents<br>3. Billing experience<br><br>The program only surveyed alumni who graduated in the last 10 years; this will be a baseline for future surveys. The program could consider sending the survey to older graduates, although the applicability to the current curriculum may not be clear.
**Annual Faculty Evaluation of Program Results**	
Faculty annual evaluation – No. of responders 59<br><br>**Strengths:**<br>1. Support and constructive feedback received from chairman and/or program director (84.75%)<br>2. Majority of faculty are involved in setting and planning and the curricula, goals and expectations for their rotations<br>3. Majority (83%) of Faculty are involved in mentoring residents<br>4. Great majority of faculty are familiar with the competencies, milestones and most ACGME regulations<br>5. Large volume and variety of cases	Overall, the feedback from the faculty is very positive regarding the residency program. There is a disconnect from this survey compared to the ACGME faculty survey; this may be due to a) a different cohort completing the survey (all faculty rather than core faculty), b) a different year with different experiences, or c) differences in how questions are phrased.<br><br>Regardless, the one area that aligns with the ACGME survey as an area of concern is "Lack of balance between clinical duties and academic teaching", where only 47% of faculty believe there is a good balance. This likely reflects the increasing volume of clinical work, and other pressures on the faculty, reducing perceived time to teach or engage in academic pursuits. Much of this is outside of the residency program's purview to change, but Dr. [redacted], Institute Chair, is

GMEC Approved 9/27/2013, 2/8/2019
Minor Edits: 4/2015, 3/2017, 1/2019

Subject/Data	Comments/Response/Plans for Improvement
6. A lot of research and academic activities opportunities<br>7. Good mix of experienced and younger staff<br>8. Dedication of program director<br>9. Comprehensive didactic lecture series and excellent educational conferences<br><br>**Weaknesses**:<br>1. Departmental educational conferences not clear for faculty<br>2. Lack of balance between clinical duties and academic teachings (only 47% perceive there is good balance). Educational activities are affected by service obligations<br>3. High service: education ratio for staff<br>4. Insufficient time for teaching<br>5. Residents lack case ownership and responsibility<br>6. Inadequate time for residents to gain good knowledge and expertise in the rotations<br><br>Comparison to last year annual evaluation:<br>Minor trends as most responses are the same when comparing the two annual evaluations.<br>1. More Faculty identify themselves as core faculty (55.9 % this year compared to 47 % last year)<br>2. More faculty report completing MedHub evaluation for the residents (77% compared to 69%)<br>3. More faculty report participating in mentoring residents (83% compared to 76%)<br>4. Only 47 % reported feeling that there is a good balance between clinical duties and academic teaching compared 60% last year<br>5. 37% reported a weakness in the ratio between service and education compared to only 25% last year.	working with the [redacted] Engagement Committee to try and improve the work experience for faculty and staff at a level that ensures all faculty have at least 5 days/month off clinical service to engage in academic pursuits. The program director will continue to advocate on the faculty's behalf on this subject.
**Annual Trainee Evaluation of Program Results**	
Resident annual evaluation – No. of responders 28<br>**Strengths:**<br>1. Great access to educational resources (100%) and respect for lecture time (96.43%)<br>2. Adequate supervision during procedures (92.86%)<br>3. Teaching during clinical activities<br>4. Promoting teamwork (100%)<br>5. Frequent and constructive feedback<br>6. Resident feeling well prepared for future career (89.29%)<br>7. Clear goals, objectives and expectations before the start of each rotation<br>8. Volume and variety of cases, combined with	The resident survey from 2019 overall had positive responses. Again, there is a disconnect with the ACGME resident survey in this regard; this may be due to a) a different cohort completing the survey, b) a different year with different experiences, or c) differences in how questions are phrased (binary vs. 5-point scale). Compared to last year's internal survey, there have been some improvements, specifically regarding more opportunity to prepare for lectures and 89% of residents feel the program provides an optimal educational environment.<br><br>Concerns raised specifically discussed in the APE included the problem of lack of continuity, follow-up on, and responsibility for cases by the residents. Improving this may help improve perceptions of faculty engagement, teaching, effectiveness of

Subject/Data	***Comments/Response/Plans for Improvement***
faculty expertise and passion to teach<br><br>**Weaknesses:** Overall, all the Reponses (> 90%) are positive, and the weaknesses mentioned here are based on the various comments that we felt are repeated throughout the response.<br>1. Education compromised by other trainees (non-residents) (35 %).<br>2. [redacted] pager interfering with didactic protected time<br>3. Lack of continuity and follow up on cases<br>4. Little training in some rotations [redacted])<br>5. Lack of adequate [redacted] and [redacted] on some occasions<br><br>Comparison to last year:<br>Overall minor trending changes.<br>1. More opportunity to prepare for lectures (17% said no now compared to 33% last year)<br>2. 89% of resident feels program provide optimal educational environment compared to 77 % last year.<br>3. 10% this year reported “No” for “is the morale good in your program” compared to 0% last year!<br>Most other areas are showing comparable results between the two years.	residents, and service vs education (by improving education). There may be misperceptions by faculty that residents are disinterested; rather, residents want the added responsibility but many faculty are reluctant to permit this. Why are the faculty reluctant?<br>1. Time pressure on staff to sign-out cases in 2 days<br>2. Concern regarding overwhelming residents (note that one day’s worth of work is so great for most faculty that they cannot be assigned days back to back – but the residents are on for 2-weeks straight).<br>3. Concern for loss of control of cases/forget about them leading to poor patient care by faculty (hard to keep track of everything).<br>How can this be solved? Disseminate faculty best practices as experienced by residents. Consider defining what “ownership of cases” looks like. What is sufficient ownership and responsibility without overwhelming residents?<br><br>The program will consider developing a task force of faculty and residents in the next academic year to further clarify these questions and issues.
**Assessment Tools (Evaluations)**	
Peer evaluation forms are still made up of binary questions instead of the Likert scale that is used for chief residents. There has been talk about making the peer evaluations more in line with the chief resident evaluations.<br><br>The [redacted] evaluations are largely the same across fields. [redacted] has a more detailed evaluation that distinctly codifies what is needed to be observed to reach a level. This detail is representative of the rotation itself. [redacted] all have the same forms. While the generic forms are serviceable some areas are more relevant to some specialties than others and make it hard for the staff and residents to have a really good idea of what is necessary to reach a level.<br><br>By March 14th, 2019, 65% of evaluations of residents have been completed with 91% on time. However, Dr. Chute had a large push for faculty to complete outstanding evaluations, and as of April 17th, 77.9% of evaluations of residents have been completed, with 52% on time. The % completion rate appears to be	All evaluations meet the ACGME Core program requirements. They are fairly easily to complete. The numerical scale-based questions range from 3-16 but the majority are between 5 and 10 questions long. All areas of the resident program are evaluated and there are many different people who provide feedback (peers, staff, technologists, and laboratory personnel). This broad range of input give the resident a good view of their performance as well as supports an interdisciplinary culture.<br><br>Evaluations are currently being updated to reflect the new milestones while still maintaining the successful qualities of the current evaluations. Some of the changes suggested are already taking place (unique evaluations for CP rotations, peer and chief evaluations aligned).<br><br>Lecture evaluations continue to be well completed and are a valuable addition to the program’s ability to adapt the curriculum and will be continued for the next academic year.

Subject/Data	Comments/Response/Plans for Improvement
hovering around 78% which is still below are goal of 80% but similar to last year (79%). We continue to see similar % completed on time (52%), as last year was only 56.7%.<br><br>The last APE had a concern about evaluation fatigue for the new lecture evaluations. It appears that most residents are filling out their lecture evaluations and the numbers have not declined over the year. The resident completion rate of any evaluation (>80%) is also consistently high throughout the year showing that these new evaluations are having little effect on the residents.	
**Case Logs/Procedures**	
The ACGME [redacted] Program Requirements state that each PGY1 resident must be directly supervised during performance of, at least, his or her three initial procedures in [redacted]. In our program, PGY1 residents are directly supervised in [redacted] for the first 6 weeks of residency by [redacted], our education [redacted]. In the 2018-2019 academic year, all of the PGY1 residents have tracked and had verified by the observer at least 3 directly supervised surgical [redacted] during July and August.<br><br>In addition, all PGY1 and PGY3 residents have a grossing competency assessment twice per year by a staff [redacted] who directly observes grossing the entire specimen. For 2018-2019, all PGY1 and PGY3 residents have passed their competencies.<br><br>To ensure the residents see an appropriate range and breadth of [redacted], PGY1 residents are asked to log at least 3 cases of the most common specimens in each [redacted] and log any rare [redacted] that they have the opportunity to [redacted]. The common requirements include a total of 22 [redacted] (total: 66 minimum cases). Thus far in the 2018-2019 academic year, the PGY1 residents who matriculated in July 2018 have logged from 35 (53%) to 64 (96%) of their expected [redacted] with 11 weeks of [redacted] remaining in the academic cycle. All current PGY2 residents logged 100% of their -required procedures in the 2017-2018 academic year, with the exception of one (who is off cycle). Residents are currently [redacted] in excess of 800 [redacted]t in the first and third years but are not required to track every case [redacted]	All graduating residents are meeting ACGME and ABP procedure requirements and seeing a broad range of surgical pathology and cytopathology cases during their training.

GMEC Approved 9/27/2013, 2/8/2019
Minor Edits: 4/2015, 3/2017, 1/2019

Annual Program Evaluation (APE)

Subject/Data	***Comments/Response/Plans for Improvement***
All of the PGY4 residents meet the [redacted] requirement of 50 [redacted] completed during their training experience. All of these [redacted] cases are documented in the ACGME case log system. All residents examine a spectrum of [redacted] cases (numbering at least 2000). All residents perform at least 200 [redacted] and examine at least 1500 [redacted] during the course of their training.<br><br>With the addition of the new [redacted], residents in [redacted] undergo a competency assessment on [redacted] cases. These are not tracked in MedHub directly, but records are kept for the purpose of [redacted].	
**Clinical Competency Committee**	
The ACGME Common Program Requirements for the Clinical Competency Committee (CCC) were reviewed. The CCC membership remains unchanged from 2017-2018 and consists of the program director ([redacted]), both associate program directors ([redacted]), and two faculty from each Department in the Institute ([redacted]). Aside from the program director, all other CCC members are core faculty. Dr [redacted] was elected Chair of the CCC for 2018-2019. Typically, three 2-hour meetings are needed to assign the 27 milestones for all 28 residents, and a fourth meeting may be held to review overall metrics or performance issues. Reviewing all residents by PGY according to a single milestone has been effective in discriminating performance levels. The CCC met on November 15, November 27, December 7, December 11, and December 18, 2018. The Spring CCC meetings are scheduled for May 22, June 4, and June 6, 2019. Fall and Spring milestone levels are entered into MedHub and WebADS by the program director and program manager in December and June each year.	The current program policy is in compliance with current ACGME requirements but will require minor edits for language to be consistent with the new Common Program Requirements (effective 7/1/2019).<br><br>The CCC will need to become familiar with metrics for Milestones 2.0 that become effective on July 1, 2019.
**Faculty Development (list activities)**	
**Cleveland Clinic Global Leadership and Learning Institute Courses attended by Professional Staff**:<br><br>20 faculty attended a total of 21 different courses (76 courses attended total).<br><br>At least one faculty attended an outside meeting related to resident mentoring and coaching.	New faculty are integrated into the program as they are hired. The faculty is knowledgeable about the ACGME competencies, milestones, fatigue and work hours.<br><br>The new mentoring program has been successful with 24 faculty participating and many resources available to mentors and mentees. We will continue to promote involvement in the mentorship program and continue to promote faculty involvement in the program and professional development

Subject/Data	*Comments/Response/Plans for Improvement*
**<u>Program-Specific Faculty Development Activities:</u>**<br><br>**Orientation:**<br><br>[redacted] met individually with 8 new faculty members to orient them to MedHub and education activities within the Institute.<br><br>**Interview Season/resident recruitment:**<br><br>One orientation session was provided by [redacted] to professional staff titled "Interviewing Tips and Allowed Questions" prior to interview season. A separate meeting was provided by [redacted] for the PGY2 residents in preparation for interview season.<br><br>**Tip of the Day series at both [redacted] and Laboratory Medicine Staff meetings:**<br><br>5 short didactics were presented in [redacted] staff meetings for a total of 10 sessions. The topics included were:<br>"Resident Retreat Feedback and Ed vs. Service",<br>"Things are Changing Around Here…",<br>"Tips for Great Teaching and Feedback",<br>"Major Changes coming to Faculty Evaluation",<br>"Self-Reflection on Teaching Improvement Exercise".<br><br>**Faculty Awareness of Key Residency-Specific Requirements:**<br>Based on the Faculty 2017-2018 Annual Survey Results:<br><br>- 88% of faculty state that they have adequate chairman support and have sufficient time in order to teach residents/fellows adequately.<br><br>- 83% actively participate in mentoring the residents/fellows in research, academic, or scholarly activity.<br><br>- 93% of faculty are familiar with the ACGME competencies, Milestones and work hour requirements.<br><br>- 80% of faculty and have participated in a group activity for improvement of the program. 85% of faculty state that they receive feedback from their chairman, and 97% state they receive feedback from residents.	activities for education. Many faculty feel like there are many professional development resources available to them but lack the necessary block time to take advantage of them. The program will continue the staff Educational Tip of the Day Series for 2019-2020, as a way of providing short, available professional development opportunities. There may be opportunities to better publicize resources available for faculty, but the method of distribution is the challenge.
**Faculty Scholarly Activity**	
The entire faculty in [redacted] had 185 unique peer-reviewed medical / scientific publications during the	Faculty scholarly activity and national reputation continue to be strengths of our program. Academic connections at other strong

Subject/Data	***Comments/Response/Plans for Improvement***
2018 calendar year. In the last year 41 out of 48 core faculty members (85%) had a published manuscript and 46 out of 48 core faculty members (96%) had a conference presentation.<br><br>In review of the core faculty scholarly activity as submitted for WebADS in August 2018, there were 209 conference presentations, 155 other presentations, and 21 textbooks/chapters that occurred or were published during the 2018 calendar year.<br><br>Our faculty members serve as editorial board members for over 50 peer-reviewed journals in the fields of [redacted]. Faculty members (core and otherwise) presented educational courses at international and national medical / scientific meetings including annual meetings for major pathology organizations such as the [redacted], and many subspecialty organizations. In addition, multiple faculty hold leadership roles in these and other organizations and serve as leaders and members of many national committees.	programs provide networking opportunities for trainees, and nearly all residents become involved in some form of scholarly activity with faculty mentors. Academic collaborations between faculty and residents contribute to optimization of fellowship matching for our residents.<br><br>In the last year, 100% of core faculty members have had a conference presentation and/or a published manuscript.
**Graduating Resident Information**	
All residents in the 2018-2019 graduating class have secured fellowships for 2019-2020:<br>• [redacted], Cleveland Clinic<br>[redacted]<br>• [redacted] Fellow, Cleveland Clinic<br>• [redacted] Fellow, Cleveland Clinic<br>• [redacted] Fellow, Cleveland Clinic<br>All but one resident in the 2017-2018 graduating class have secured "first real job" or additional fellowships for 2019-2020:<br>• [redacted] Atlanta, GA<br>• [redacted], Columbus, OH<br>• [redacted] – Assistant Professor, University of Utah/ARUP<br>• [redacted] Fellow, Cleveland Clinic<br>• [redacted] Fellow, Loyola U, Chicago, IL<br>• [redacted] Fellow,	Our residents are successful in obtaining competitive fellowship positions, both at our institution and around the country. Our alumni are successful in obtained "real jobs" after fellowship completion and show continued academic productivity after leaving our residency program.

Subject/Data	Comments/Response/Plans for Improvement
Cleveland Clinic<br>• [redacted] – Currently seeking opportunities<br>Graduates have shown continued participation in scholarly activities<br>• 2018 graduates have 8 publications post-graduation<br>• 2017 graduates have 7 publications post-graduation<br>• 2016 graduates have 15 publications post-graduation	
**Interprofessional Education**	
Inter-professional education (IPE) refers to occasions when students from two or more professions learn together with the object of collaborative practice for patient-centered healthcare.<br><br>Our residents learn alongside of [redacted] fellows in the areas of [redacted].<br>Interactions with fellows are variable, depending upon the clerkship with fellows often acting as additional educators and mentors.<br><br>Our residents interact regularly with residents and fellows from other departments with a good example being regular and repeated communications with trainees in dermatology. In this setting, [redacted] residents impart knowledge about [redacted] and biology while [redacted] colleagues enhance learning regarding clinical settings and therapeutic options.<br><br>[redacted] residents on [redacted] clerkships regularly interact with students engaged in clinical clerkships in the both [redacted] School and in the School of [redacted]. In these interactions, our residents are often teachers in "real-world" settings pertaining to patient care and diagnostics.<br><br>These are a few examples of IPE in our program.	The Cleveland Clinic residents in [redacted] experience IPE in many settings during their four years of training.
**Major Changes in the Program since last ADS update**	
There has been no change in leadership in our Institute or Program this academic year.<br><br>This year the revised Formal Education Curriculum (Didactics/Seminars) was implemented, including a	This first year has served as a pilot for the new curriculum and evaluation system. Many lectures have been revised to be more interactive. A [redacted] Boot camp was created and very popular. Residents regularly complete evaluations and are able to commend good lectures and point out lectures with perceived

Subject/Data	*Comments/Response/Plans for Improvement*
new [redacted] boot camp. With this, new lecture evaluations were created and implemented to accompany the new series. Included in this new curriculum was a Quality/Patient Safety Curriculum.<br><br>A friendly competition was created to encourage residents to engage in educational activities, called the [redacted].<br><br>Some faculty turnover has occurred [redacted] have joined as faculty within the last year. Faculty departures include [redacted].<br><br>All Program Letters of Agreement were reviewed, and no new sites were added this year.	decreased educational value in order to simultaneously adapt teaching strategies from exceptional lectures and target areas for improvement. The feedback has been generally favorable, with some specific recommendations for improvement in the lecture series for next year.<br><br>The [redacted] has fostered engagement in both the lecture series and in the weekly nationwide Children's Pediatric [redacted] Case of the Week, as well as the [redacted] case of the week. A winning team will be declared in June, and awards will be distributed. The committee felt this was a positive experience and will be continued for the next year.
**Outside Rotators/Other Learners**	
The total number of outside rotators has slightly increased from 133 (2017-2018) to 142 (2018-2019).<br><br>The number of CWRU medical students has increased from 14 (2017-2018) to 35 (2018-2019). On the other hand, the number of CCLCM students has dropped from 22 (2017-2018) to 14 (2018-2019).<br><br>This year new rotations were available for students and visitors. This included [redacted] (previously available in 2016-2017).<br><br>The number of rotating residents (CCF and non-CCF), fellows, medical students outside CWRU And CCLCM and high school students are relatively similar to the last two academic years.<br><br>The number of international visitors slightly increased from 10-12. They are a diverse group from around the world.<br><br>A subset of residents felt that their education has been somehow affected by the presence of outside rotators (30% in 2018; 36% in 2019). The most common reasons include:<br>- Inability to ask questions in a relaxed environment and generate resident-level discussion<br>- Space limitations in sign-out rooms<br>- Large number of observers rotating at the same	There has been a slight increase in outside rotators over the last academic year, largely due to an increase in CWRU medical students rotating in our department, which is perceived largely as a positive, as there is a strong need to recruit good physicians to enter our profession.<br><br>There continue to be concerns by some residents (36%) regarding education compromised by other learners. There is particular concern regarding space issues related to the upcoming renovations in L2, as well as the creation of new fellowships and expansion of existing fellowships. The program will need to work with the Educational Vice Chair and Institute Chair to ensure that this expansion and renovation does not compromise resident access to cases, previewing, and learning.<br><br>Last year a major change was implemented to reduce the length of time [redacted] residents rotate on [redacted], as this was the rotation with the greatest dissatisfaction by the residents with interference by other learners. This change has been successful.<br><br>Other rotations that by report are most affected include:<br>• [redacted] – likely due to rotators entering from both [redacted]; issue is mainly previewing time, not during sign-out. Possible options include limiting rotators, creating more preview space. This will be discussed with the [redacted] service director and education coordinator at the annual May update.<br>• [redacted] – issues regarding sign-out room use by fellow, residents unable to take cases

Subject/Data	*Comments/Response/Plans for Improvement*
time<br>- Medical students are rarely interested in contrast to most fellows<br>-	from room, less heads on the microscope. Options include reduced restrictions on case movement, fellow using assigned cubicle instead. This will be discussed with the [redacted] service director and education coordinator in the annual May update.
**Outstanding Program Accomplishments/Kudos**	
For the fifth year in a row the residency program was ranked in the top ten nationally in the US News and World Report/Doximity Survey and was one of only 3 programs at Cleveland Clinic to rank in the top ten within a specialty ([redacted]).<br><br>The program received continued accreditation from ACGME at its January 2019 RC meeting. One citation (service vs. education) was extended and three previous areas for improvement/concerning trends were dropped.<br><br>Program representatives ([redacted] attended the Howard University Residency Fair on February 23, 2019 as part of our Diversity Outreach Plan to recruit minority medical students. We were one of two [redacted] residency programs to attend.<br><br>A group of residents mentored by [redacted] won the first-place ribbon at the Cleveland Clinic's Second Annual GME Patient Safety Day- *A Day of Interprofessional Learning* on March 15, 2019. The project, [redacted]<br><br>In addition, the following residents received local/national awards:<br><br>[redacted]:<br>Runner-up Abstract Award<br>[redacted]<br><br>[redacted]:<br>Best Resident Poster Award<br>[redacted]<br>Resident Leadership Institute Scholarship<br>[redacted]<br><br>[redacted]:<br>[redacted] Resident Travel Award<br>Housestaff Association Art Contest, 1st Place Photography	The program has had a successful year and winning the first prize in GME patient safety day was a highlight.

Subject/Data	*Comments/Response/Plans for Improvement*
John Beach Hazard Faculty Teaching Awards:<br>[redacted]<br><br>George C. Hoffman Teaching Award:<br>[redacted]	
**Program Goals & Objectives**	
Our [redacted] residency program at Cleveland Clinic is different from other training programs in several ways. Our trainees experience exceptionally large volumes of cases in [redacted]. These cases range from "bread and butter" to esoterica. Our program is also a genuine mix of patient care focused work and scholarly pursuits with approximately half of graduates pursuing community practice positions and the other half entering academia. The alternating [redacted] annual structure of the program is a unique approach as well. Our program is special in the diversity of residents from many countries around the globe. We differentiate ourselves from other programs by encouraging but not demanding research involvement, by providing funds for travel and books, and facilitating one on one experiences with world thought leaders in many pathology subspecialty areas.	We recommend no changes to the current residency program goals.
**Program Match Results/Recruitment Efforts**	
**Match Results**: The program matched 7/7 positions offered through the NRMP. The mean/median USMLE Step 1 and Step 2 CK scores for all matched applicants were 250/253 and 254/252, respectively. For comparison, scores in 2018 were 242/243 and 251/251, respectively. Including the new applicants, we will have 12 men and 16 women in our residency program.<br><br>**Applications:** The program received 561 applications (124 US/437 IMG) through ERAS that included 67 allopathic seniors (MD) and 33 osteopathic seniors (DO). Nationally, 33% of all US allopathic seniors who ranked a [redacted] in NRMP applied to our program this year. We invited 103 candidates (70 USG/33 IMG) to interview and interviewed 84 (53 USG/31 IMG). The interview acceptance rate was 82%; 19 applicants invited did not schedule or cancelled an interview. Reasons applicants gave for cancelling an interview included time, expense, or travel problems. Our program continues to attract top applicants from around the country, with most from the Midwest (Ohio – 9, Michigan – 7, Pennsylvania – 5, New York – 4, Missouri – 3, Illinois – 2, Oklahoma – 2,	The program was very successful in matching 7 top applicants through the NRMP Match this year, despite a decrease in applicants nationally. Our program continues to attract top applicants from around the country, with most from the Midwest.<br><br>The top reasons for applying to the Cleveland Clinic remain similar to last year and include reputation, fellowship opportunities, referral by a friend or advisor, and online ranking by Doximity.<br><br>The biggest barriers to recruiting residents remain 1: negative perceptions of living in Cleveland; 2: proximity of location to family and friends, and 3: local environment, including the cold.<br><br>Review of the interview season mechanics shows the small-group experience is positive, and a similar structure will be continued for 2019-2020.<br><br>Overall costs for recruitment were up 9% over the prior year; this was largely due to increased costs for dinner and lunch. The program will continue to encourage PGY2s to take out candidates to good restaurants to ensure a good experience, but to consider the budget when choosing the location.

Annual Program Evaluation (APE)

Subject/Data	*Comments/Response/Plans for Improvement*
Wisconsin – 2, Virginia – 2, Indiana – 1, North Dakota – 1, and Tennessee – 1). Nationally, 33% USG entering [redacted] applied to our program.<br><br>**Comparison to Prior Year:** Nationally, total applications to [redacted] decreased by 48 (3%) compared to last year, with 1,554 total applicants in 2018 and 1,506 total applicants in 2019. Nationally, there were 32 fewer USG and 16 fewer IMG. Applications to our program decreased by 42 (8%), with 12 fewer USG and 30 fewer IMG. However, this decrease reflects the national decrease in applications to pathology.<br><br>**Recruitment:** Interviews were scheduled on 24 days from October 24-January 14, and the itinerary for the interview day remained unchanged from last year. The program provided a night's stay at a nearby hotel and provided gift bags (bag, O'Malley's chocolate bar, Humphrey Popcorn ball, and Cleveland Clinic merchandise chosen by the marketing department (pens, magnet, water bottle, notepad, earphones, etc.)). The program also sent a New Year's holiday card. One applicant had a positive comment about the "warm, heartfelt holiday card." The number of candidates per day ranged from 1-4. The program director interviewed almost all candidates, with the exception of 4 candidates on one interview day, and 33 faculty participated in interviews. The total expense for interview dinners was $5,155 (up from $4,655 last year), with an additional $2,304 spent for box lunches (up from $1,483 last year), and $7,304 spent for hotel accommodations (similar to $7,412 last year). The Cleveland Clinic merchandise was donated to the program from the [redacted] Marketing department (thank you [redacted]) and the candy bars and popcorn balls were donated to the program by [redacted].	Providing hotel has become an expectation for many applicants, and we are grateful for the support of [redacted] to permit funding of hotel for our applicants.
**Quality Improvement and Patient Safety Projects (include status)**	
All senior residents were required to participate in at least one mock CAP inspection in their final two years.<br><br>All residents are now part of a Quality/Patient safety (QPS) project.<br>• PGY2-4 completed their quality project and presented at QPS Grand rounds.<br>• PGY1-3 residents were assigned to one of three new quality projects selected by the program director and QPS Chief Resident.<br><br>To date, 15 of 27 residents completed the GME Resident/Fellow Quality improvement and Patient	With the implementation of mandatory involvement in QPS projects, all residents are now reporting involvement with a project for the first time.<br><br>We have met our prior goals with all projects being presented and finished.<br><br>The Quality/Patient Safety Chief Resident position has been continued for the 2019-2020 academic year. [redacted] will meet with the 2018-2019 QPS Chief to discuss opportunities for improvement in the QPS curriculum for next year.

Annual Program Evaluation (APE)

Subject/Data	*Comments/Response/Plans for Improvement*
Safety Self-evaluation. The survey provided information for the July-April timeframe.<br>Highlights:<br>• 93% of the residents report that CCF promotes a just culture of safety always or most of the time.<br>• 80% of the residents believe that shared concerns/reports about quality and safety issues lead to improvements in patient care<br>• 100% of residents report that they can report QPS concerns without fear of retribution.<br>• 100% of residents report involvement in a QPS project in the last year.<br><br>All quality projects were accepted at the Cleveland Clinic Patient Safety day. One project won first prize.<br><br>At least one project's abstract was accepted for a national meeting. Other abstracts are submitted pending acceptance<br><br>Two residents serve on the PLMI safety committee. All PGY-2 and PGY-4 residents sat on at least one quality committee this year.<br><br>23 SERS events were reported by fellows/residents for the first (13) and second quarter (10) of the academic year. This was in the top 10 programs of over 40 residency/fellowship programs at CCF.<br><br>New QPS Educational Curriculum including:<br>• Lectures<br>• Online modules<br>• Mock Root cause analysis<br>• Test Utilization simulation<br>• Proficiency Testing simulation<br>• Pre and Post QPS Exam	
**Research Curriculum**	
A survey was sent to the current residents to assess their current research training and future needs. The results were as follows:<br>- The great majority (more than 94%) of the residents have participated in at least one project during their residency so far, and almost all of them have at least one poster &/or manuscript accepted for publication (average 3.47 per resident)<br>- 66% of them reported being involved in designing the project<br>- Current formal training in research the residents have is based on onboarding didactic training, online CITI training. Informal training through practice and being involved in active projects.<br>- About half the residents are interested in having	While there is not a formal research curriculum in the program, many resources are available, and most residents engage in research throughout their time in the program.<br><br>There may be opportunities to create an optional additional curriculum for residents who are interested; this can be discussed with [redacted], who is the Vice Chair for Research.

Subject/Data	Comments/Response/Plans for Improvement
a more dedicated research training incorporated into the curriculum, while the other half prefer the practical way if learning through experience and being involve in more research projects.<br>- Majority (90%) of the residents wants more opportunities to be involved in research, and willing to commit extra time for it.<br>- Around 57% report using published articles during clinical work/ study and around 78% believe they have the tools to critically evaluate the studies they use.<br>- Residents report Journal club (in 2nd year) and discussion with faculty during sign - out to be the two main ways they learn about evaluating and critically think about the research studies they read.	
**Results of last year's Program Improvement Plan (PIP)**	
The most recent PIP in 2018 was reviewed. The PIP identified multiple areas of strength including 100% resident completion rate of internal and ACGME surveys, [redacted] identified as a great investment by the residents and part of excellent access to educational resources, resident and staff acknowledgement that the program accepts feedback and has been acting upon recommendations with a trend to improvement, many research opportunities and mentorship, compliance with ACGME requirements to provide rotation objectives, work hour compliance, and evaluations, and high volume and diversity of cases.<br><br>Multiple issues were examined including clinical teaching (high workload), didactics (lecture quality), service versus education ([redacted], and quality and patient safety (resident involvement in projects).<br><br>**Service versus education:** The workload over the past several years has increased for both faculty and residents. Reducing the service versus education conflict has been a priority for the program. The frequency of on-call blood bank pages is being monitored at each monthly education meeting and has decreased significantly since changes were implemented. The [redacted] service constitutes critically important work and does not have a "[redacted]" support person available. We are in the process of attempting to secure a dedicated [redacted] for the [redacted] rotation – the position has been approved, but additional staffing needs in the [redacted] [redacted] have taken priority. Nearly 43% of residents rate the [redacted]	Most of the areas of strength and concern identified in the PIP process are similar to those identified in the program's APE process. The program is making good progress in many areas, but there are others that still need to improve. For full details, see the results of the APE from this year and last year.

Subject/Data	Comments/Response/Plans for Improvement
rotation as good/outstanding.<br><br>**Quality and patient safety projects:** The program implemented group QPS projects, and the current schedule ensures that all residents are involved in a QPS project every year. QI projects are mandatory for residents. Annual group QPS projects are supported by the Institute and presented at PLMI Grand Rounds in December, as well as the annual Cleveland Clinic GME Patient Safety Day Fair. The new QPS curriculum implemented for the 2018-2019 academic year is composed of self-study modules and hands-on practice exercises with faculty. Lastly, the position of QPS Chief Resident was created to help engage residents and faculty in QPS.<br><br>**Clinical teaching:** Surgical services have been divided into teaching and non-teaching services to decrease case volume to increase faculty time for teaching. Recruitment of additional faculty is an ongoing priority. The program director began an "Educational Tip of the Day" series presented at monthly [redacted] staff meetings, and this will be continued in the upcoming academic year.<br><br>**Didactics:** A new didactics schedule was started in July 2018, in addition to an 8-week [redacted] boot camp series in July and August. The program director has advocated for more interactive and case-based teaching approaches. The early didactic sessions have been well received by the residents.<br><br>**Implement a dedicated pediatric pathology curriculum:** All residents are currently subscribed to the Nationwide Children's Pediatric [redacted] Case of the Week. The Cleveland Clinic has purchased a subscription for Pediatric [redacted] 2019 [redacted] online which provides 6 [redacted] cases on a quarterly basis [redacted] is compiling a master pediatric [redacted] study set for residents. Additionally, the possibility of implementing a mandatory pediatric [redacted] rotation at an outside institution is being explored.	
**Review of last year's Annual Program Evaluation (APE)**	
See table below.	
**Trainee Performance on Board Exam (if applicable)**	
The [redacted]) examinations are held in Spring and Fall of each year. In Spring 2018, 6 of 7 graduates took the examination for the first time, all in [redacted]. The comparative statistics for first time takers (CCF vs national) for that exam are:	For the last 5 years, our program graduates continue to exceed the national pass rates in [redacted] for first time takers. Over the range from 2012-2018, 98% of residents have passed both [redacted] portions of the board exam on the first attempt; only one resident did not pass both portions of the exam on the first

Subject/Data	***Comments/Response/Plans for Improvement***
**CCF Pass Rate (2018) National Pass Rate (2018)**<br>[redacted]<br>6/6 (100%) 6/6 (100%) 92% 94%<br><br>**CCF Pass Rate (2012-2018) National Pass Rate (2012-2018)**<br>[redacted]<br>55/56 (98%) 53/54 (98%) 92% 94%<br><br>Based on a rolling five-year average, our graduates ranked in the upper third for all topical areas in the [redacted] exams. All but one graduate completing the program from 2012-2018 has taken the exam.	chance but did pass on the second attempt.<br><br>The program is exceeding expectations for board performance metrics: at least 80% of graduates from the preceding 5 years take the exam; at least 70% of graduates from the preceding 5 years pass the exam on the first attempt.<br><br>The graduates' performance on the [redacted] exams has been in the upper third for all topical areas, suggesting that the program curriculum and learning opportunities are strong preparation for the board exam.
**Trainee Performance on In-Service Exam (if applicable)**	
The [redacted] RISE examination was administered in a proctored setting on March 25-26, 2019. The six-hour examination consists of approximately 360 multiple choice questions covering all areas [redacted]. Overall performance for PGY1-PGY4 residents was just above the national mean at 504 vs. 493, which placed the program at the 60th percentile nationally.<br><br>By class, the average score (and national mean score) were as follows: PGY1: 462 (449); PGY2: 495 (482); PGY3: 519 (508) and PGY4: 546 (542). Generally, residents are performing better in areas that corresponded to their field of focus that year, i.e. they tend to perform better in cytopathology and surgical pathology if they are on [redacted], and similarly in [redacted] areas once they are on [redacted] years.<br><br>Areas that CCF residents were significantly (65th percentile overall or higher) above their peers in other programs included: [redacted].<br><br>Areas of weakness (overall below the 50th percentile) included: [redacted].	The program shows consistent performance above the national mean on the RISE exam over the last 5 years, with an expected correlation of improved scores in the areas they are rotating each year. Compared to the ABP exam, our residents appear to perform better on the board exam than the RISE exam. This is likely due to encouragement by the program not to study for the RISE exam and use it largely as a self-assessment tool.
**Trainee Scholarly Activity**	
In the last academic year, since July 1st, 2018, 15 out of 27 residents (56%) have been involved in presentations at national meetings (range: 1-4), with a department total of 30 presentations.<br><br>In the last academic year, there were 13 published	Scholarly activity by our trainees remains a strong point of the program.<br><br>The most common meetings over the last 4 years for national presentations include the:<br>[redacted] (29

Subject/Data	Comments/Response/Plans for Improvement
manuscripts (range: 1-2) authored by 9 residents (33%).<br><br>Of the graduating residents, during the entire training period, 4/6 (67%) had at least 1 published manuscript (range 1-4) and 4/6 (67%) had at least 1 national presentation (range 5-9) during residency training.<br><br>A list of 2018-2019 publications for all residents and graduating seniors is attached.	presentations),<br>[redacted] (23 presentations),<br>[redacted] (5 presentations),<br>[redacted] (5 presentations),<br>[redacted] (2 presentations),<br>[redacted] (1 presentation).
**Well-Being**	
Steps within the residency program to promote well-being of residents include:<br>• Attention to work hours<br>• Education about signs of fatigue and offering call rooms for naps and/or reimbursement for an Uber home<br>• Promotion of the Caring for Caregivers program which offers free mental health services and wellness resources<br>• Periodic Wellness Grand Rounds<br>• Random Acts of Kindness committee which is a resident run committee that holds periodic events to allow residents and faculty to decompress<br>• Membership to the Walker Fitness center<br>• Periodic events hosted by the Program Directors to promote camaraderie within the residency program<br>• GME intranet page with numerous wellness resources<br>• Three weeks of vacation time with few restrictions on when and how it may be used, in addition to five leave of absence days<br>• A leave of absence day designated for personal health care<br>• Flexibility and willingness to find coverage for emergencies<br><br>Steps within the department to promote faculty well-being include:<br>• Promotion of the Caring for Caregivers program which offers free mental health services and wellness resources<br>• Periodic Wellness Grand Rounds<br>• Random Acts of Kindness committee which is a resident run committee that holds periodic events to allow residents and faculty to decompress<br>• Membership to the Walker Fitness center<br>• One "personal" day a year<br>• Vacation and meeting time off service	There is a large focus on wellness within the department and residency program. [redacted] is commended for his extensive work in securing reliable call rooms for the residents this year.<br><br>A high percentage of residents and faculty scored all questions on the ACGME well-being survey between three and five and few scored any of the questions as one or two, which is similar to the national means.<br><br>A possible area for improvement is educating both residents and staff on who to report to when something tragic happens at work. This could be discussed with residents at each 6-month evaluation meeting to reinforce periodically.

Subject/Data	Comments/Response/Plans for Improvement
• GME intranet page with numerous wellness resources<br><br>Based on ACGME well-being data from the 2017-2018 resident survey 74% of residents reported sometimes, often, or very often reflecting on how their work makes the world a better place. 88.8% felt vitality to do their work sometimes, often, or very often. 96.3% felt supported by their coworkers sometimes, often or very often. 88.9% were sometimes, often, or very often proud of their work and 77.1% were sometimes, often, or very often eager to return to work. All residents reported feeling as though their basic needs are met sometimes, often, or very often. 77.7% feel connected to their work sometimes, often or very often. 96.3% feel the amount of work they are expected to complete in a day was sometimes, often, or very often reasonable and 88.8% feel they participate in decision that affect their work sometimes, often, or very often. 59% stated that they often or very often know who to call when something tragic happened at work and 37% felt this was not applicable.<br><br>Based on ACGME well-being data from the 2017-2018 faculty survey 88.4% of faculty reported sometimes, often, or very often reflecting on how their work makes the world a better place. 93% sometimes, often or very often felt vitality to do their work and 97.7% felt supported by coworkers sometimes, often or very often. All faculty reported feeling proud of their work sometimes, often or very often and 88.5% sometimes, often or very often felt eager to return to work the next day. 97.7% felt their basic needs are met sometimes, often, or very often and 97.6% felt they ate well sometimes, often, or very often. 79.1% felt the amount of work they are expected to complete in a day is reasonable sometimes, often or very often. 83.8% reported participation in decisions affecting their work sometimes, often or very often. 37.3% stated that they often or very often know who to call when something tragic happened at work and 58.1% felt this was not applicable.<br><br>Survey numbers appear to be comparable to national means other than enjoyable interactions with patients, which are not applicable for most of us, and knowing who to call when something tragic happens.	
**Work Hours**	
From July 1st, 2018 through March 10th, 2019, there have been 2 work hour violations (see below). The average number of hours recorded per week by the	The program is highly compliant with ACGME work hour requirements. The number of work hour violations has decreased from the prior year. Work hour violations are rare (n

Subject/Data	Comments/Response/Plans for Improvement
residents is 48.7 hrs/week. The maximum hours per week has a range of 62.5 hrs/week to 81.5 hrs/week (after removing outliers from Blocks 7 & 9, discussed previously). The rotations with the longest work hours vary, but often include [redacted] (15.5 - 16.5 hours), [redacted] (15 – 17 hours), [redacted] (16 - 18 hours), and [redacted] (15 – 16 hours).<br><br>The program coordinator reminds residents to record work hours each week; overall compliance with work hour recording is excellent, with 96.4-100% of work hour timesheets submitted each block and 93-100% submitted on time.<br>In the new call system designed this year, PGY1 residents have "practice" call with PGY3 supervision and duties ending at 10 PM, until January 1. Weekend autopsy calls are taken by PGY1 residents with PGY3 supervision. Starting January 1, PGY1 residents take independent weekday overnight calls and weekend autopsy calls, and PGY3 residents take weekend AP call. The PGY3 residents appreciate having calls more spaced out after January 1, with call roughly every 2 weeks. Even when supervising between July and December, PGY1 residents are quick to acclimatize and the workload is a lot less for PGY3 residents.<br><br>Two residents are moonlighting, volunteering to perform [redacted] after hours. Moonlighting work hours are being tracked appropriately through MedHub.<br><br>*Block 1: Both violations were of the "8-hour rest" rule by two separate PGY3 residents. Both residents were on-call that night in addition to wrapping up [redacted] cases from a prior weekend. One resident left at 11:30 PM and returned to preview at 7 AM with 7.5 hours between shifts. The other resident left at midnight and returned to work at 6 AM to prep [redacted] cases and preview, with 6 hours between shifts. [redacted] discussed with both residents the circumstances of these days, as well as the options if they are too fatigued to return to work the next day.<br><br>*Blocks 7 & 9: Of note, while it is not a work hour violation, [redacted] met with one resident regarding the high number of hours submitted, as they are an outlier. In discussion, this was an error in entering work hours as the resident was incorrectly submitting "working" hours for the full 24 hours of taking [redacted] call, not adjusting for the actual time spent answering clinical calls.	= 2) and are reviewed monthly by the Education Committee. The longest work hours (for individual days, data not shown here) are logged by PGY1 and PGY3 residents on anatomic pathology rotations. The new AP call system is working well for both PGY1 and PGY3 residents.<br><br>There may be an opportunity for the new PGY1s to learn about efficiency early on in the program, to prevent issues. A "survival tips/Efficiencies learned" talk by a senior PGY3 resident may help.

Blocks 1-6	1*	2	3	4	5	6
Ave Hours	48	50.1	50.2	48.8	48.3	47.8

Annual Program Evaluation (APE)

Subject/Data	Comments/Response/Plans for Improvement

Ave Hours (Excluding home call)	46	47.9	48	48.5	48	47.5
Av days off	2.1	1.9	1.8	1.9	2	2.1
24+4	100%	100 %	100%	100%	100 %	100 %
8 hr rest	98.5 %*	100 %	100%	100%	100 %	100 %
14 hr rest/24 hr call	100%	100 %	100%	100%	100 %	100 %
Max hrs/week	81.5	63	67	65.5	67.5	62.5
Max ave hrs	63.4	61.6	60.6	62.1	58.8	57.2
Submission Rate	100%	99.3 %	96.4%	100%	100 %	100 %

7-13	7*	8	9*	10	11	12	13
Ave Hours	45.7	49.2	50.4	48.6	49.5	48.6	NA
Ave Hours (Excluding home call)	43.2	46.6	49.6	48.3	48.9	48.1	NA
Av days off	2.4	2.1	1.9	1.9	2.0	2.1	NA
24+4	100 %	100 %	100 %	100 %	100 %	100 %	NA
8 hr rest	100 %	100 %	100 %	100 %	100 %	100 %	NA
14 hr rest/24 hr call	100 %	100 %	100 %	100 %	100 %	100 %	NA
Max hrs/week	91*	67	95*	69	68	70	NA
Max ave hrs	65.3	57.6	66.3	58	65.6	60.8	NA
Submission Rate	100 %	97.1 %	97.1 %	98.5 %	97%	97%	NA

Additional Discussion Items (add/remove lines as necessary)

Parameter	Comments/Response/Plans for Improvement
**ACGME Milestone Assessments**	
Milestone assignments from Spring 2018 and Fall 2018 were reviewed and compared to national metrics for pathology from ACGME. The 2018-2019 graduating class of PGY4 residents achieved level 4 or higher in 11/27 milestones (41%) for Spring 2018 and 10/27 milestones (37%) for Fall 2018.<br><br>For PGY4 residents, milestone range was level 3.6 – 4.1 for Spring 2018. Level 3.79 or higher was achieved in 26/27 milestones (96%) for Spring 2018.<br><br>The majority of our residents achieve at least level 3.79 by graduation in nearly all patient care, medical knowledge,	Overall, graduating PGY4 residents are largely successful in achieving the expected level 4 of all milestones. There is a clear progression in development from PGY1 to PGY4 in all Milestones over the 4 years of residency.<br><br>The System Based Practice Milestones remain the most difficult to assess, and the most difficult to achieve level 4 during residency, but this is similar to the national data.<br><br>To help improve the SBP milestone achievements, we have implemented the [redacted] Curriculum. As part of this initiative, residents are required to complete [redacted] University, participate in mock inspections,

Parameter	***Comments/Response/Plans for Improvement***
professionalism, and interpersonal and communication skills milestones.<br><br>The System Based Practice Milestones remain the most difficult to assess, and the most difficult to achieve level 4 during residency. The most problematic include:<br>○ Lab Management – resource utilization: personnel and finance (SBP3), PGY4 peer average level 3.8<br>○ Lab Management – technology assessment (SBP6), PGY4 peer average level 3.6<br>○ Lab Management – Informatics (SBP7), PGY4 peer average level 3.8<br><br>When compared to the 2018 ACGME milestone summary report, SBP3, SBP6, and SBP7 appear to be universally difficult categories in which to achieve and average of 4 nationally.<br><br>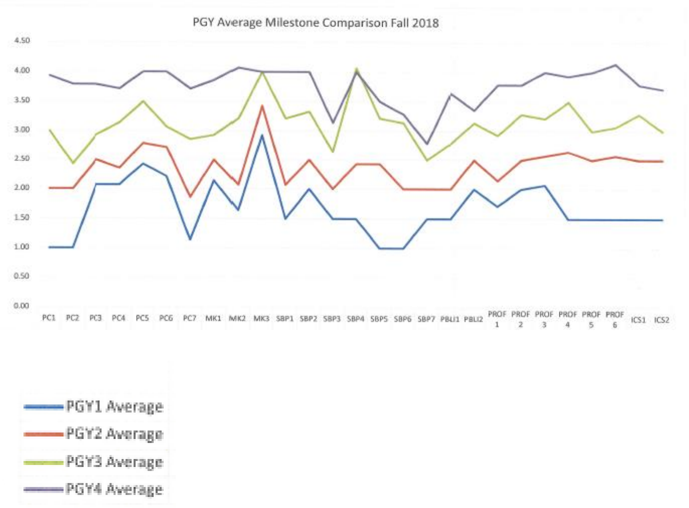	complete a root cause analysis module, and be active in group quality improvement projects.
**[redacted] Rotations**	
Rotation evaluations sent (n= 140) and completed (n=128) during July 1, 2018 to April 30, 2019 were reviewed and included required and elective rotations for each section. Evaluations of most [redacted] rotations are favorable, with overall ratings of Outstanding, Good or Satisfactory exceeding 80% in all rotations except CP Cross Cover (75%). There was concern (>10% responses) regarding "volume/workload (not) appropriate" in [redacted] and "(not) appropriate balance of service vs education" [redacted].	Feedback on rotation evaluation comments and suggestions for improvement will be discussed with the faculty in each Section in May 2019.<br><br>Ongoing efforts to adjust service vs. education include: 4 PM cutoff for [redacted] cases; attempting to balance resident and fellow numbers on [redacted] and [redacted]; and decreasing number of calls in [redacted].

Parameter	Comments/Response/Plans for Improvement
[redacted chart]<br><br>Excerpted comments pertinent to each rotation include:<br><br>• [redacted]: Strengths- "discussions on [redacted]" "got to choose modules of particular interest to me"; Weaknesses- "sign-out at different locations" "[redacted] worksheets" "modules and reading materials need to be updated".<br>• [redacted]: Strengths: "daily mini sessions" "high volume of cases"; Weaknesses: "coding" "volume of cases in afternoon".<br>• [redacted]: Strengths: "faculty" "sign-out" "seeing interesting and educational cases"; Weaknesses: "workload, especially late afternoon reference lab flows".<br>• [redacted]: Strengths: "sign-outs" "variety of cases, knowledgeable faculty" "more resident autonomy"; Weaknesses: "tracking down [redacted] and cases to be presented at tumor board"<br>• [redacted]: Strengths: "rounds" "outstanding staff" "balance of clinical responsibilities and education"; Weaknesses: "assignment to [redacted] with same workflow".<br>• [redacted]: Strengths: "TRIG" "diversity of cases and teaching"; Weaknesses: "none".<br>• [redacted]: Strengths: "A lot of clinical exposure" "[redacted] week" "faculty" "learning how to handle [redacted] calls"; Weaknesses: "volume and service work" "calling patients who are scheduled for surgery" "constant paging during rounds" "too few residents" "service vs. education"<br>• [redacted]: Strengths "willingness of staff to include resident" "time for [redacted]"; Weaknesses: none.	
**Anatomic Pathology Rotations**	
[redacted chart]<br><br>Overall notes:<br>- Overall performance ratings range from 3.28 – 4.88 out of 5.<br>- Faculty are always available, passionate and engaged in residents' teaching throughout the rotation.<br>- Great variety of educational cases is one of the main strengths of all rotations.<br>- Great hands – on [redacted] experience with complex cases in all rotations (frozen section, autopsy and surgical pathology rotations)<br>- Most individual evaluation parameters are above 4.5 (out of 5) in almost all rotations; except the presence of adequate workspace which underperforming in [redacted] and [redacted]	Feedback on rotation evaluation comments and suggestions for improvement will be discussed with the faculty in each Section in May 2019. Best practices will be discussed to assess for areas for continued advancement of each rotation.<br><br>Many of the issues seen in the rotation evaluations are part of larger issues for the overall program. The rotations with the greatest areas for improvement include:<br>• [redacted] – still awaiting a dedicated [redacted] to assist at this location, as recruitment and hiring has been a problem. Once a [redacted] has been hired, it will be important to create defined expectations, goals and objectives for each sequential rotation residents take. A new [redacted] Service Director was recently announced, [redacted].<br>• [redacted] – issues related to service vs. education

Parameter	Comments/Response/Plans for Improvement
[redacted])<br><br>Best practices from high performing rotations:<br>- Structured, well oriented curriculum (including assessment tool and pre-arranged teaching sessions) ([redacted])<br>- Ample available resources and study sets ([redacted])<br>- More time previewing the cases and reading on them ([redacted])<br>- Have a great fellow passionate and engaging in residents' education while on service ([redacted])<br>- Great and Adequate feedback ([redacted])<br><br>Notes from underperforming rotations:<br>- Busy rotation with no time to have an educational experience with the cases ([redacted])<br>- Lack of clear educational expectations from the rotation ([redacted])<br>- Carry over service work can affect education on other services ([redacted])<br>- Learning from high Yield complex cases is hindered and lost with the load of cases being presented ([redacted])	will be discussed with the faculty and service director, to explore opportunities to improve the experience.
**Conferences/Didactic Sessions**	
On a weekly basis, residents are mandated to attend four didactic morning lectures (two [redacted] and two [redacted]) and one Grand Rounds from 8-9 AM. Lectures that were historically delivered between 7-8 AM are now given from 8-9 AM. Conferences during the months of July and August are exclusively dedicated to introductory sessions that mandate attendance for PGY-1 and PGY3 for AP and PGY2 and PGY4 for CP. Additionally, [redacted] conference is held twice a week and is mandatory for PGY1 residents. A new 2-year based lecture series plus [redacted] boot camp have started this academic year (2018-2019). Additionally, lecture evaluations are now delivered to residents who attended directly after the lecture is over, which will help modifying the curriculum based on residents overall satisfaction.<br><br>As of mid-March, there have been:<br>• [redacted] conferences: 39 introductory sessions plus 73 lectures, 17 of which are [redacted] seminars.<br>• [redacted] conferences: 34 introductory ([redacted]) sessions (new) plus 72 lectures, 9 of which are [redacted] based seminars.<br><br>**Conflict with service obligations:**<br>While the 8-9 AM slot every day is resident protected	The conference schedule was extensively changed this past year and has been an overwhelming success. These changes have been very positively received by faculty and residents. This system will continue going forward.<br><br>The number of unknown seminars has increased this year, as well as the number of external visiting professors. The program has ensured VPs were invited and presented on topics that are needed by the program ([redacted], informatics) have been included this year.<br><br>The Housestaff Seminar Series (new this year) has been successful, although there may be opportunities to improve the day/time (possibly Mondays at noon) for better attendance by residents and faculty. This will be explored to determine if there is availability of space and faculty for a new time.

Parameter	*Comments/Response/Plans for Improvement*
didactic time, there are a few interferences:<br>[redacted]: The resident can get paged out of lecture.<br>**AP rotations:** Residents cannot preview part of the material for the 9AM sign out, especially in high-volume rotations and/or when cases are delivered from [redacted] after 8 AM and some of the busier [redacted] rotations will occasionally interfere with lecture.<br><br>**Conference attendance rate**<br>PGY1: 88.5% (meeting expectation) vs. 78.7% for 2017-2018<br>PGY2: 77.8% (meeting expectation) vs. 75.7% for 2017-2018<br>PGY3: 80.8% (meeting expectation) vs. 72.8% for 2017-2018<br>PGY4: 82.7% (meeting expectation) vs. 43.25% for 2017-2018<br><br>**Visiting professors/ guest lecturer for this academic year and topics covered:**<br>[redacted], Ohio State University, Columbus, OH, GU [redacted] (Advancing Diagnostic [redacted])<br>[redacted], Case Western Revere University, OH, [redacted] Informatics ([redacted])<br>[redacted], University of Michigan, Breast Pathology ([redacted])<br>[redacted], University of Michigan, Clinical Chemistry ([redacted])<br>[redacted], Memorial Sloan Kettering Cancer Center, Director of Experimental [redacted] ([redacted])<br>[redacted], Children's Hospital of Philadelphia, [redacted]<br>[redacted], Case Western Reserve University School of Dental Medicine, [redacted]<br>[redacted], University of Washington, [redacted]<br>[redacted], Chief [redacted]<br>[redacted], University of Michigan [redacted]<br><br>**Housestaff Seminar Series:**<br>This year, a new Housestaff seminar series was developed to create a scholarly presentation requirement for all residents and fellows. This series is currently presented	

Annual Program Evaluation (APE)

Parameter	*Comments/Response/Plans for Improvement*
every other Wednesday from 1-2pm from mid-August through June. It has been a very important activity but has shown declining attendance over the course of the year despite [redacted] encouragement of attendance by faculty. The residents find the time slot challenging to attend due to service obligations. The topic and speakers could also be better advertised.	

Results of last 2 year's Annual Program Evaluation Action Plan(s)

	Areas for Improvement (AY 2017 - 18)	Intervention	Date instituted / Individual responsible	Expected Resolution (outcome measures and date)	Status (resolved, partially resolved and detail, not resolved and date)
1	**Service vs. Education Conflicts**	1. Optimize the new [redacted] rotation through implementation of [redacted] at the [redacted] desk, and redistributed work responsibilities; [redacted]<br>2. Monitor [redacted] pages during the day and on call for residents at monthly education meetings and provide feedback to the [redacted] faculty; [redacted]<br>3. Survey residents anonymously regarding additional areas of imbalance in service vs. education for future interventions and discussion of what is considered non-physician work and what services they feel that it is excessive; [redacted] and chief residents	[redacted]	Overall Goal: Improvement on ACGME resident survey by 10 points over 2017 survey<br>1. Goal of >70% of residents rate the FS rotation as Good or Outstanding on the rotation evaluation by May 2019; pre/post surveys of the resident's and faculty's perception of the [redacted] rotation show improvement in satisfaction<br>2. Goal of continued sustained reduction of [redacted] pages, staying at the current level of approximately 400/month, 7/night on call on average<br>3. Goal of at least 70% of residents respond to survey by July 1st, 2018, to be developed by [redacted] and chief residents	**Partially Resolved. Goal Not Achieved. Citation Continued.**<br>Overall Goal: 2018 ACGME survey did not change on this metric (41% vs. 41%).<br><br>1. Only 44% of residents rate the [redacted] rotation as either good or outstanding. This does not meet the >70% desired threshold. The anticipated hiring of a [redacted] dedicated [redacted] has not happened.<br><br>2. Average number of TM pages per month is 422 with an average of 6 call per night on call. These numbers are acceptable.<br><br>3. 100% of eligible residents responded to the survey.
2	**Quality and Patient Safety Projects**	1. Continue group QPS projects aligned with institute goals for 2018-2019, soliciting ideas from faculty/chairs for new projects to start in Jan 2019; [redacted] and the new QPS chief<br>2. Focus on completion of 2018 QPS projects with required presentation at a [redacted] QPS Grand Rounds in fall, 2018 Dr. Chute and the new QPS chief<br>3. Require group and individual 2018 QPS projects to be presented at the Cleveland Clinic QPS Day in March 2019 [redacted] and the new QPS chief<br>4. Roll out the new QPS curriculum	[redacted], QPS chief resident	Overall Goal: 100% of residents involved in a QPS project on the ACGME survey and internal QPS surveys in 2018-2019.<br>1. 100% PGY1-3 residents portfolio document involvement in a quality project (independent or group) in 2018-2019<br>2. 100% of 2018 projects completed by January 2019<br>3. At least 3 QPS project posters from [redacted] presented at the QPS day in March, 2019	**Resolved**<br>Due to new requirement that all residents participate in a QPS project, all residents are now involved with a quality project. This has been confirmed by the Internal QPS survey.<br><br>All QPS projects started in early 2018 were completed and presented at [redacted] QPS Grand Rounds<br><br>All 2018 projects were

GMEC Approved 9/27/2013, 2/8/2019
Minor Edits: 4/2015, 3/2017, 1/2019

		in 2018-2019.			accepted and presented at Cleveland clinic Patient Safety day in March 2019.
3	**Faculty Engagement**	1. Continue mentorship program, and encourage new faculty to join as mentors, and increase faculty development on mentorship (encourage faculty to take the staff mentorship and coaching workshop, distribute educational materials through shared drive. [redacted]<br>2. Continue the Educational Tip of the Day Series at staff meetings, focusing on innovative teaching styles, feedback skills, and evaluation completion; [redacted]<br>3. Identify additional ways to recognize faculty for their teaching and program contributions by surveying faculty and the chair; [redacted]<br>4. Advocate to the Department and Institute Chair for additional resources or new efficiencies to provide faculty with more time to teach successfully; [redacted]<br>5. Work to provide faculty with increased feedback on their performance through compiled MedHub evaluation data with Program Director notes at least once per year, along with conference evaluation data upon request; [redacted]	[redacted]	Overall Goal: Increase ACGME resident survey responses regarding faculty interest in education and creating an environment of inquiry by 10% compared to 2017 survey<br>1. At least 25 faculty participating in the mentorship program, and all residents who want a faculty mentor have one (per discussion in 6-month evaluation with program director)<br>2. At least 6 Educational Tip of the Day presentations at staff meetings in 2018-2019<br>3. Faculty ACGME survey results show an increase in "sufficient time to supervise residents" by 10% from 2017 survey<br>4. Faculty ACGME survey results show an increase in "faculty satisfied with personal performance feedback" by 10% from 2017 survey	**Resolved**<br>**Area for Improvement Removed**<br>Overall Goal: 2018 ACGME survey showed no significant change, however, the 2019 ACGME survey show at 10%+ increase in "sufficient instruction" 59% to 89%, and "faculty and staff create environment of inquiry" from 56% to 70%.<br><br>1. 24 residents with 24 faculty participating in the mentorship program, all residents paired successfully with a mentor.<br><br>2. To date, 5 Educational Tips of the Day have been presented since April 2018.<br><br>3.Faculty ACGME survey results show a 10% increase from 74% (2017) to 84% (2018) (maintained at 80% for 2019) for "sufficient time to supervise residents".<br><br>4. Faculty ACGME survey results show a 16% increase from 65% (2017) to 81% (2018) (maintained at 78% for 2019) for "faculty satisfied with personal performance feedback"
4	**Pediatric Pathology Education**	1. Investigate options for an online curriculum for [redacted] available for purchase for the residents; Currently examining the option of a subscription to the Society [redacted]<br>2. Create and distribute [redacted]<br>[redacted] Continue resident subscription to	[redacted]	Overall Goal: Improved perception by graduating seniors regarding [redacted] training on the ACGME survey, with a score of over 3.5<br>1. At least [redacted] completed and available in the resident area for review at all times by November 2018	**Partially Resolved**<br>**Goal Not Achieved but Area for Improvement Removed**<br>Overall Goal: 2018 ACGME Survey Score was 2.4 (not at goal).<br><br>1. The [redacted] is now completed and available to all residents (approximately 25

		the [redacted] of the Week from Ohio State with public recognition of residents who get the answer correct; [redacted]<br>4. Increase the number of pediatric [redacted] lectures in the morning curriculum series; [redacted]		2. Have at least 2 external speakers [redacted] topics in the Grand Rounds series in 2019-2019<br>3. Have at least 4 talks on [redacted] topics in the 8am lecture series in 2018-2019	cases). [redacted] creation is ongoing.<br><br>2. Three external speakers on [redacted] have presented in the Grand Rounds series thus far in 2018-2019.<br><br>3. The 8 a.m. lecture series for 2018-2019 has included 5 talks on [redacted].
**Areas for Improvement (AY 2016 - 17)**		**Intervention**	**Date instituted / Individual responsible**	**Expected Resolution (outcome measures and date)**	**Status (resolved, partially resolved and detail, not resolved and date)**
1	**Recognize and increase faculty engagement in resident education**	Encourage faculty engagement in resident education through mentorship program, random acts of kindness committee (RAK), staff meeting Educational tips of the day.	NA	Track participation in mentoring program by residents and faculty. Compare outcomes of resident and faculty surveys (2018 internal and 2018 ACGME surveys). Review Institute scores on annual Press Ganey caregiver experience survey.	**See 2017-2018 APE resolution status**
2	**Improve quality and timeliness of resident evaluation by faculty.**	Report to Education Committee and Department Chairs. Regular reminders to faculty regarding evaluation completion. Educational tips of the day on how to complete evaluations in MedHub efficiently/ effectively with good feedback.	NA	Track completion rate by rotation block. Goal: 80% completed, 60% on time.	**Partially resolved:**<br>**2019 Update**: 77.9% of evaluations of residents have been completed, with 52% on time. This is similar to last year.
3	**Reduce conflicts between service and education.**	Advocate to [redacted] leadership to hire an additional [redacted] for the [redacted] service. Track pages to the [redacted] Pager monthly, reported to the Education Committee.	NA	Ongoing monitoring of blood bank pages. Results of rotation surveys for [redacted] and informal resident feedback.	**See 2017-2018 APE resolution status**
4	**Re-evaluate/re-design the didactic conference series.**	Convene a group of faculty and residents to review conference offerings and offer a proposal for 2018-2019 conferences by March 2018.	NA	Implement a new didactic curriculum staring July 1st, 2018, with plan created and approved by March 2018.	**Resolved:**<br>A task force was created, and recommendations were made for the restructuring of the didactic conference series.<br><br>New lecture evaluations are in the process of being created.

5	**Every resident involved in QI/Patient Safety (PS) projects.**	Involve all residents in QPS projects through implementation of mandatory selected group projects each year of residency.	NA	Documentation in MedHub via Learning Modules, with progress tracked in portfolio. Semi-annual GME survey results.	**Resolved:**<br>All PGY1-3 residents are now involved in a PS/QI project of their own creation or have been assigned to one of three projects chosen by the Program Director as of January 2018.

This Year's Annual Program Evaluation Action Plan(s)

Area for Improvement Identified	**Intervention**	**Date to be instituted/ Individual responsible**	**Expected Resolution (outcome measures and date by)**
**Service Vs. Education**	1. [redacted]:<br>Plan A: Recruitment and retention of [redacted], clear delineation of responsibilities and expectations by year/rotation.<br>Plan B: Work with faculty to improve experience until plan A occurs, continue [redacted] support for lunch (as available). Options include disseminating best practices, dedicated educational time.<br>[redacted]: Continued work to maintain or further reduce calls to residents when on [redacted]<br>3. [redacted]: Work with [redacted] faculty to determine ways to optimize resident experience on [redacted]	[redacted]<br>3. [redacted]	**Overall Goal:** Elimination of ACGME [redacted] for Service Vs. Education in 2020<br>**Secondary goal:** 2019-2020 ACGME resident survey improves by at least 10 points on service vs. education<br>**Tertiary Goals**<br>1. Improvement on [redacted] rotation evaluation by at least 10% satisfactory or better<br>2. Improvement on [redacted] rotation evaluation by at least 10% satisfactory or better<br>3. Average number of [redacted] pages per month remains similar or reduced to 2018-2019 data
**Resident Graduated Responsibility and Case Follow Through**	1. Create [redacted] Task Force composed of faculty and residents examine ways of aligning expectations and improving responsibility<br>2. Look for opportunities in [redacted] for additional opportunities with graduated responsibility	[redacted]<br>2. [redacted]	**Overall Goals:**<br>1. 2019-2020 Faculty ACGME survey shows improvement in perception of effectiveness of graduated residents, and internal GME survey shows reduced comments that residents take ownership of cases.<br>2. Resident internal GME survey shows improved experience with continuity of cases.<br>**Interim Goal:** Task force creates actionable recommendations to be implemented in 2020-2021 academic year.
**Space planning to accommodate all learners**	Anticipating new fellowship programs in development/starting, there is need to ensure adequate space for all learners.<br>1. Purchase new microscopes and [redacted] for fellows in [redacted] cubicle	[redacted]<br>4.	**Overall Goal:**<br>Improvement in internal GME resident survey for question "do other learners interfere with education."

	space and encourage use of this area to maximize other available preview spaces for residents/other observers<br>2. Work with space planning to ensure renovations being planned for [redacted] will include adequate preview space and appropriate space in [redacted]<br>3. Work to develop additional preview space in the interim for residents/observers prior to renovations start<br>4. Work with faculty on various service to ensure ready access to cases for previewing	[redacted]	**Interim goals:**<br>1. Purchase new [redacted] and [redacted] for all new fellowship programs starting in July 2019, and purchase a dictaphone for all existing fellows without a one by end of July 2019.<br>2. [redacted] will meet with all [redacted] subspecialty directors/education coordinators to discuss fellows/resident preview location and come up with tentative plan.<br>3. Create at least one additional preview space by July 2019 in current [redacted] area.

Which aspects of Health Systems Science were incorporated into your curriculum during this academic year? *(Place an X in left column to denote; describe in right column)*

	Subject Area	***Description***
■	Clinical Informatics	Three lectures on pathology informatics are included in our [redacted] Lecture Series.
☐	Health Care Delivery System	
■	Health Care Policy & Economics	We have two lectures per year on health care and laboratory finance given by [redacted], who is the National [redacted] Representative to [redacted] committee to the Centers for Medicare & Medicaid Services.
☐	Leadership in Healthcare	
■	Patient Safety	All residents are involved in Quality/Patient Safety projects in PGY1-3 and will likely complete 3 or more over the course of their residency. In addition, we have multiple lectures and learning modules on Quality and Patient Safety as part of our curriculum, including Modules on Handoffs, and Error Management (IHI modules on errors, culture of safety, and SERS).
■	Population Health	On the [redacted] rotation, residents are exposed to current population health issues related to [redacted], including tracking, monitoring and reporting of public health issues (most recently the opioid epidemic). This includes directed reading and direct experience with reporting.
■	Quality Improvement	All residents are involved in Quality/Patient Safety projects in PGY1-3 and will likely complete 3 or more over the course of their residency. In addition, we have multiple lectures and learning modules on Quality and Patient Safety as part of our curriculum, including Modules on Process Improvement (root cause analysis, Plan Do Check Act, 5S, test utilization) and [redacted]), along with mandatory participation on [redacted] committees for a minimum of 3 months.
☐	Socio-ecologic Determinants	

■	Teamwork & Team Science	Our learning module on Handoffs explores the issues of teamwork and high vs. low performing teams and the barriers that teams need to overcome to provide the best patient care.
☐	Use of Assessments to Support Learning Improvement	
☐	Value in Health Care	

SWOT ANALYSIS - This will assist your program on preparing for the Self-Study, as you will need to use past APEs to develop a comprehensive summary of the program and its learning environment. A SWOT Guide and SWOT Analysis Template are located on GME|com.

Strengths	Weaknesses
• Large volume and variety of cases in nearly all subspecialties and services<br>• Advanced laboratory with excellent technological facilities for testing<br>• Mix of common and complex case mix<br>• National/international expert faculty with subspecialty focus<br>• Many opportunities for research and funded for multiple national meeting presentations<br>• Many fellowship opportunities<br>• Many educational resources provided (book fund, [redacted], education only meetings, etc.)<br>• Institute Chair with strong interests in supporting education<br>• All rotations located on single campus (Main Campus)	• Continued issues of service vs. education imbalance<br>○ Busy/overwhelming workload (residents and faculty)<br>○ Greatest areas: [redacted]<br>• Complex system of case distribution/completion and turnaround time pressure that leads to challenges including<br>○ lack of follow-up on cases for residents (ancillary testing, etc)<br>○ Giving residents responsibility in way faculty and residents want<br>• Large department/many faculty, and faculty traveling to additional hospital sites, resulting in limited connections between faculty and residents, leading to engagement issues<br>• [redacted] curriculum (lack of subspecialty sign-out)<br>• Outdated [redacted]<br>• Many trainees (residents/fellows/medical students) /observers complicating preview space/access to cases
**Opportunities**	**Threats**
• New [redacted] – consider additional resident position for [redacted] only or [redacted] only with research focus, possibly partnered with LRI<br>• Diversity and size of program/department leads to networking opportunities (jobs/alumni feedback, etc) – could we develop a better alumni network/job posting for alumni?<br>• GME/GLLI resources for faculty and resident development around feedback, quality/patient safety<br>• Possible pediatric pathology away rotation at Rainbow Babies<br>• Increased opportunities for resident involvement in [redacted]<br>• Increased opportunities for resident involvement in committees for national [redacted] organizations and local organizations (i.e. Cleveland Clinic Housestaff association)<br>• Additional fellowship development to complement learning environment for residents<br>• Numerous faculty engaged in academic pursuits creating opportunities for resident involvement<br>• Excellent Cleveland Clinic	• Constant increase in volume due to CC growth and acquisition of new hospitals<br>• Era of “do more with less” pressure on RVU per faculty reducing availability for teaching/engagement<br>• New fellowship program development and balance with residency training<br>• Funding (additional resident spot, hotel for applicants, resources for education, support staff FTE’s for administration, etc)<br>• Other programs with integrated curriculum as more attractive/desirable? More flexibility needed?<br>• Challenges in recruiting/hiring/retaining mid-level providers (i.e. [redacted]) to help balance service vs. education

Annual Program Evaluation (APE)

Program Director Attestation – Typed signature below indicates that the APE content, or a synthesis of the APE, was reviewed and distributed to the program faculty:

[redacted]
6/11/2019

Please refer to the ACGME Common Program Requirement V.C. Program Evaluation and Improvement for additional information regarding the requirement.

Documentation to be used for the Annual Program Evaluation

- ■ Review and current status of Action Items identified in the last Annual Program Evaluation
- ■ Program Goals and Objectives
- ■ Assessment of previous curriculum changes
- ■ Assessment tools (evaluations)
- ■ Faculty development activities/needs:
  - ☐ Summary of faculty evaluations–development needs, areas for improvement
  - ■ Scholarly activities
- ■ Recruitment and retention of faculty and trainees
- ■ Trainee match results
- ☐ Patient satisfaction surveys (if applicable)
- ■ Results of annual CC trainee program evaluation
- ■ Results of annual CC faculty program evaluation
- ■ Results of ACGME Resident Survey
- ■ Results of ACGME Faculty Survey
- ■ Results of previous Program Improvement Plan (PIP)
- ■ Previous RRC Notification Letters or Communications
- ■ Trainee performance:
  - ■ Certification exams
  - ■ Aggregate data from formative assessments
  - ■ In-service exam scores
  - ■ Scholarly activities
  - ■ Alumni surveys (*when available)*
- ☐ Other: ____
