# Supplementary material for: Best Practices for Building and Supporting Effective ACGME-Mandated Program Evaluation Committees
Source: MedEdPORTAL. 2020 Dec 10;16:11039. doi: 10.15766/mep_2374-8265.11039 (PMC7732133; doi:10.15766/mep_2374-8265.11039)
Supplement: Supplementary file 1 — Facilitator Guide for PEC Workshop.docxPEC Best Practices Presentation.pptActivity 1 Pair-and-Share.docxActivity 2 Small-Group Discussion of Aims.docxActivity 3 Small-Group Discussion of Data Sources.docxAPE Weak Example.pdfAPE Strong Example.pdfAPE Template With Notes.docSession Evaluation Form.docx [file mep_2374-8265.11039-s001.zip › I. Session Evaluation Form.docx]

**Session:** Best Practices for ACGME-Mandated Program Evaluation Committees

**Date & Time:** [insert date and time]

**SESSION OBJECTIVES**

| **Were the following session objectives met?** | **MET** | **NOT MET** |
| --- | --- | --- |
| Demonstrate understanding of Program Evaluation Committees for program improvement and ACGME accreditation. |  |  |
| Discuss role of Program Evaluation Committee members and others in monitoring and documenting training program activities and outcomes. |  |  |
| Discuss best practices of highly functioning Program Evaluation Committees. |  |  |

**SESSION ORGANIZATION**

|  | | **STRONGLY**  **DISAGREE** | **DISAGREE** | **AGREE** | **STRONGLY**  **AGREE** |
| --- | --- | --- | --- | --- | --- |
| The amount of material presented was appropriate given the length of the session. | |  |  |  |  |
| A safe learning environment allowed for asking questions, making comments, and sharing opinions. | |  |  |  |  |
| Interactive teaching strategies were used (think-pair-share; small group discussions; large group discussions) | |  |  |  |  |
| The examples presented in the session aided in my understanding of appropriate ….. | |  |  |  |  |
| The templates provided are useful and will be a valuable resource in the future. | |  |  |  |  |
| Consulting and discussing with colleagues from other departments was meaningful and helpful. | |  |  |  |  |
| **What was the most useful portion of this session?** |  | | | | |
| **What is one thing we should make sure to repeat in future sessions?** |  | | | | |
| **What suggestions can you offer to help improve this session?** |  | | | | |

**DEMOGRAPHICS**

| **Primary role:**  ▢ Program Director  ▢ Associate Program Director  ▢ Program Coordinator  ▢ Other: _______________________________________ | **Time in current role:**  ▢ Preparing for first-time role in program  ▢ Preparing to transition to new role in program  ▢ First year in this role  ▢ Other: _______________________________________ |
| --- | --- |
