# Supplementary material for: Best Practices for Building and Supporting Effective ACGME-Mandated Program Evaluation Committees
Source: MedEdPORTAL. 2020 Dec 10;16:11039. doi: 10.15766/mep_2374-8265.11039 (PMC7732133; doi:10.15766/mep_2374-8265.11039)
Supplement: Supplementary file 1 — Facilitator Guide for PEC Workshop.docxPEC Best Practices Presentation.pptActivity 1 Pair-and-Share.docxActivity 2 Small-Group Discussion of Aims.docxActivity 3 Small-Group Discussion of Data Sources.docxAPE Weak Example.pdfAPE Strong Example.pdfAPE Template With Notes.docSession Evaluation Form.docx [file mep_2374-8265.11039-s001.zip › H. APE Template with Notes.doc]

**Program Information (all information available in MedHub and ADS (ACGME site))**

| Academic Year |  |
| --- | --- |
| Program Name |  |
| Program Director |  |
| Percentage of Time PD dedicates to this program |  |
| Associate Program Director(s) |  |
| Percentage of Time APD(s) dedicates to this program |  |
| Program Coordinator |  |
| Percentage of Time PC dedicates to this program |  |
| Date of Last RC Site Visit/Self-Study Visit |  |
| Date of Upcoming Self-Study Visit |  |
| Current Accreditation Status |  |
| Current Number of Citations |  |
| Current Number of Area of Improvement/Concerning Trend |  |
| Length of Program |  |
| # of Trainees per Year |  |
| Total # of Approved RC Positions |  |
| Total # of Approved CCF Positions |  |
| Program Evaluation Committee (PEC) Members |  |
| Date(s) of PEC Meeting(s) held this academic year |  |
| Date of Annual Program Evaluation (APE) |  |
| Date APE and Action Plan was reviewed and approved by Faculty |  |

**Attendees at the Annual Program Evaluation Meeting (add/remove lines as necessary)**

| ***Name*** | ***Title*** |
| --- | --- |
|  |  |
|  |  |
|  |  |
|  |  |
|  |  |
|  |  |
|  |  |
|  |  |
|  |  |

**What are the aims of this training program - what differentiates this program from others?**

**Describe activities taken this academic year to further the aim**

**Annual Program Evaluation Discussion Items (Constructed during APE meeting)**

| ***Subject*** | ***Parameter*** | ***Comments/Response/Plans for Improvement*** |
| --- | --- | --- |
| ACGME Annual Faculty Survey Results  (available in MedHub- program accreditation section) |  |  |
| ACGME Annual Resident Survey Results  (available in MedHub- program accreditation section) |  |  |
| ACGME Milestone Update  (available in ADS) |  |  |
| ACGME RC Notifications & Reponses (if applicable)  (available in MedHub- program accreditation section and ADS) |  |  |
| Alumni Survey Results (if applicable)  (individual program results- internally tracked or reference alumni office) |  |  |
| Annual Faculty Evaluation of Program Results  (available in MedHub- program accreditation section) |  |  |
| Annual Trainee Evaluation of Program Results  (available in MedHub- program accreditation section) |  |  |
| Assessment Tools (Evaluations)  (available in MedHub) |  |  |
| Case Logs/Procedures  (available in MedHub- reports or ADS case log system) |  |  |
| Clinical Competency Committee  (available in MedHub, if manually entered in system already; reference internal program notes/meeting minutes) |  |  |
| Faculty Development (list activities) |  |  |
| Faculty Scholarly Activity |  |  |
| Graduating Resident Information  (individual program results- internally tracked or reference alumni office) |  |  |
| Interprofessional Education |  |  |
| Major Changes in the Program since last ADS update |  |  |
| Outside Rotators/Other Learners  (available in MedHub- schedules) |  |  |
| Outstanding Program Accomplishments/Kudos  (individual program information) |  |  |
| Program Goals & Objectives  (available in MedHub) |  |  |
| Program Match Results/Recruitment Efforts |  |  |
| Quality Improvement and Patient Safety Projects (include status)  (information available internally and/or MedHub) |  |  |
| Research Curriculum |  |  |
| Results of last year’s Program Improvement Plan (PIP) |  |  |
| Review of last year’s Annual Program Evaluation (APE) |  |  |
| Trainee Performance on Board Exam (if applicable) |  |  |
| Trainee Performance on In Service Exam (if applicable) |  |  |
| Trainee Scholarly Activity |  |  |
| Well-Being |  |  |
| Work Hours  (available in MedHub- reports) |  |  |

**Additional Discussion Items (add/remove lines as necessary)**

| ***Parameter*** | ***Comments/Response/Plans for Improvement*** |
| --- | --- |
|  |  |
|  |  |
|  |  |
|  |  |
|  |  |

**Results of last 2 year’s Annual Program Evaluation Action Plan(s) (MedHub)**

| **Areas for Improvement (AY 20XX - XX)** | | **Intervention** | **Date instituted/ Individual responsible** | **Expected Resolution (outcome measures and date)** | **Status (resolved, partially resolved and detail, not resolved and date)** |
| --- | --- | --- | --- | --- | --- |
| 1 |  |  |  |  |  |
| 2 |  |  |  |  |  |
| 3 |  |  |  |  |  |
| …. |  |  |  |  |  |
| **Areas for Improvement (AY 20XX - XX)** | | **Intervention** | **Date instituted/ Individual responsible** | **Expected Resolution (outcome measures and date)** | **Status (resolved, partially resolved and detail, not resolved and date)** |
| 1 |  |  |  |  |  |
| 2 |  |  |  |  |  |
| 3 |  |  |  |  |  |
| …. |  |  |  |  |  |

**This Year’s Annual Program Evaluation Action Plan(s)**

| ***Area for Improvement Identified*** | ***Monitoring & Measuring Plan*** |
| --- | --- |
|  |  |
|  |  |
|  |  |
|  |  |
|  |  |

***Which aspects of Health Systems Science were incorporated into your curriculum during this academic year? (Place an X in left column to denote; describe in right column)***

| ***Subject Area*** | | ***Description*** |
| --- | --- | --- |
| **🞎** | Clinical Informatics |  |
| **🞎** | Health Care Delivery System |  |
| **🞎** | Health Care Policy & Economics |  |
| **🞎** | Leadership in Healthcare |  |
| **🞎** | Patient Safety |  |
| **🞎** | Population Health |  |
| **🞎** | Quality Improvement |  |
| **🞎** | Socio-ecologic Determinants |  |
| **🞎** | Teamwork & Team Science |  |
| **🞎** | Use of Assessments to Support Learning Improvement |  |
| **🞎** | Value in Health Care |  |

**SWOT ANALYSIS - This will assist your program on preparing for the Self-Study, as you will need to use past APEs to develop a comprehensive summary of the program and its learning environment. A SWOT Guide and SWOT Analysis Template are located on** [**GME|com**](http://portals.ccf.org/gme/Sections/Program-Directors-Coordinators/Accreditation/ACGME-Annual-Program-Review)**.**

| **Strengths (internal, positive)** | **Weaknesses (internal, negative)** |
| --- | --- |
| - What advantages does your program have? - What does your program do better than anyone else? - What do caregivers in your program see as your strengths? - Examples may include: strong educational support, engaged/diverse teaching faculty, etc. | - What could your program improve? - What are people in your program likely to see as weaknesses? - Are like programs doing something better than yours? - Where do you feel there are gaps/shortcomings within the program? - Examples may include: feedback on resident/fellow evaluations, resident/fellow involvement in Quality and Patient Safety (QPS), etc. |
| **Opportunities (external, positive)** | **Threats (external, negative)** |
| - What opportunities can you spot? - What are the current ongoing trends within the program (positive and/or negative) that can develop opportunities for the program? - Changes in technology, policies, etc. - Examples may include: utilizing available resources for faculty development, developing a QPS curriculum, etc. | - What obstacles does your program face outside of your control? - Could any of your weaknesses seriously threaten your program? - What is your competition doing that might cause difficulties for your program? - Examples may include: funding, resources, hospital growth, etc. |

Program Director Attestation – Typed signature below indicates that the APE content, or a synthesis of the APE, was reviewed and distributed to the program faculty:

(PD Name) (dated)

Please refer to the ACGME Common Program Requirement V.C. Program Evaluation and Improvement for additional information regarding the requirement.

***Documentation to be used for the Annual Program Evaluation***

🞎	Review and current status of Action Items identified in the last Annual Program Evaluation

🞎	Program Goals and Objectives

🞎	Assessment of previous curriculum changes

🞎	Assessment tools (evaluations)

🞎	Faculty development activities/needs:

- Summary of faculty evaluations–development needs, areas for improvement
- Scholarly activities

🞎	Recruitment and retention of faculty and trainees

🞎	Trainee match results

🞎	Patient satisfaction surveys (if applicable)

🞎	Results of annual CC trainee program evaluation

🞎	Results of annual CC faculty program evaluation

🞎	Results of ACGME Resident Survey

🞎	Results of ACGME Faculty Survey

🞎	Results of previous Program Improvement Plan (PIP)

🞎	Previous RRC Notification Letters or Communications

🞎	Trainee performance:

- Certification exams
- Aggregate data from formative assessments
- In-service exam scores
- Scholarly activities
- Alumni surveys (*when available)*
- Other:__________________________________________________________________
